# Clinical and Prognostic Significance of *CEBPA* Mutations in Myelodysplastic Syndromes

**DOI:** 10.3390/cancers18132135

**Published:** 2026-07-01

**Authors:** Mohamed M. Khamis, Aref Al-Kali, Omar Alkharabsheh, Aleksandar Babic, Ranju Kunwor

**Affiliations:** 1 Department of Medicine, Mercy Hospital St. Louis, St. Louis, MO 63141, USA; 2 Division of Hematology, Mayo Clinic, Rochester, MN 55905, USA; 3 Division of Hematology/Oncology, University of Cincinnati College of Medicine, Cincinnati, OH 45227, USA; 4 Hematology/Oncology, Blood and Marrow and Cellular Therapy Program, SSM Health Saint Louis University Hospital, St. Louis, MO 63104, USA

**Keywords:** myelodysplastic syndromes, *CEBPA*, prognosis, leukemic transformation, IPSS-R, truncating mutations, bZIP domain

## Abstract

**Background/Objectives:** Myelodysplastic syndromes (MDS) carry a highly variable prognosis, stratified by the Revised International Prognostic Scoring System (IPSS-R) and the Molecular IPSS (IPSS-M). *CEBPA* mutations define a favorable-risk subgroup in acute myeloid leukemia (AML), yet their prognostic significance in MDS has not been characterized. **Methods:** We analyzed 2442 patients from the International Working Group (IWG) 2022 multi-center MDS registry after pre-specified exclusions. Overall survival (OS) and leukemia-free survival (LFS) were compared between *CEBPA*-mutated (*n* = 66; 2.7%) and wild-type patients using Kaplan–Meier estimation and Cox proportional hazards regression, adjusting for age, sex, and IPSS-R score; pre-specified subgroup, sensitivity, competing-risk, and mutation subtype analyses were performed. **Results:** *CEBPA*-mutated patients had markedly inferior OS (median 17.2 versus 42.2 months; HR 2.05, 95% CI 1.50–2.79; *p* < 0.001). After IPSS-R adjustment, the hazard ratio remained adverse (HR 1.39, 95% CI 1.00–1.94; *p* = 0.053), with uniform directionality across all 13 evaluable subgroups and no significant interaction. Co-mutation adjustment for *ASXL1* and *STAG2* further attenuated the hazard ratio to HR 1.11 (95% CI 0.79–1.57; *p* = 0.54), suggesting part of the observed signal reflects co-mutation burden rather than an independent *CEBPA* effect. Competing-risk analysis suggested that the excess mortality is mediated through AML transformation (*CEBPA*-mutated versus wild-type subdistribution hazard ratio of 1.89, 95% CI 1.20–2.99; *p* = 0.006) rather than non-transformative MDS mortality (cause-specific HR 0.97; *p* = 0.890). Truncating mutations drove the adverse signal (HR 2.21; *p* = 0.023), while basic leucine zipper (bZIP) domain mutations showed no significant effect (HR 1.25; *p* = 0.470). **Conclusions:** *CEBPA* mutations identify a rare MDS subgroup with markedly inferior survival, driven by truncating loss-of-function mutations and associated with leukemic transformation; the AML-derived bZIP-favorable paradigm does not translate to MDS, and *CEBPA* mutation status merits a prospective study to assess clinical utility for risk stratification.

## Simple Summary

Myelodysplastic syndromes are blood cancers in which the bone marrow does not make healthy blood cells well. Some patients later develop acute leukemia, but it is difficult to know who is at the highest risk. This study focused on changes in the CEBPA gene, which are considered favorable in some patients with acute leukemia. We asked whether the same idea applies to patients with myelodysplastic syndromes. Using a large international patient registry, we found that CEBPA changes were uncommon but linked to shorter survival and a higher chance of leukemia transformation. The poor outcome was strongest in patients with gene changes that are likely to stop normal CEBPA function. These patients also often had other high-risk gene changes. Our findings suggest that CEBPA changes should not be viewed as favorable in myelodysplastic syndromes. They may help identify patients who need closer monitoring and future studies of earlier treatment.

## 1. Introduction

Myelodysplastic syndromes (MDS) are clonal disorders of hematopoiesis with pre-leukemic potential, characterized by ineffective hematopoiesis, peripheral cytopenias, and a variable risk of transformation to acute myeloid leukemia (AML), with a median overall survival ranging from less than six months in very-high-risk disease to more than eight years in very-low-risk disease, with AML transformation occurring in 15–30% of patients during follow-up [1,2]. The Revised International Prognostic Scoring System (IPSS-R), incorporating the bone marrow blast percentage, cytogenetic risk group, and severity of three cytopenias, is the primary framework for risk stratification and treatment decision-making [3]. However, the IPSS-R does not incorporate somatic mutation data, which are now recognized as independently prognostic determinants of survival in MDS.

Systematic genomic profiling has identified mutations in *ASXL1*, *RUNX1*, *TP53*, and *EZH2*, and spliceosome-pathway genes (*SF3B1*, *SRSF2*, *U2AF1*), among others, as independent prognostic determinants beyond the IPSS-R [4,5,6,7,8,9]. The Molecular International Prognostic Scoring System (IPSS-M), developed by Bernard and colleagues using the IWG 2022 multi-center registry, integrates 31 molecular variables with clinical parameters to generate a continuous risk score with substantially improved prognostic discrimination relative to the IPSS-R [10]. The independent prognostic contributions of the individual genes contributing to the IPSS-M residual mutation term (Nres) have not been separately characterized.

*CEBPA* encodes C/EBPα, a crucial transcription factor for granulocytic commitment during normal myeloid differentiation [11]. In AML, biallelic *CEBPA* mutations, especially bZIP in-frame insertions, are classified as favorable risk in the ELN 2022. Five-year overall survival rates approach 60% after intensive induction chemotherapy, reflecting unique chemosensitivity and a co-mutational landscape, with few additional mutations at diagnosis [12,13,14]. The WHO 2022 5th edition continues to use a ≥20% blast threshold for diagnosing AML with *CEBPA* mutations, owing to limited evidence to lower this cutoff [15]; consequently, patients with fewer than 20% blasts are categorized as MDS and are typically treated with hypomethylating agents rather than intensive chemotherapy. This approach differs from *NPM1*-mutated AML, where intensive chemotherapy is used regardless of the blast count, reflecting established chemosensitivity. Whether this treatment–response analogy extends to *CEBPA* mutations in MDS, where the treatment context and co-mutations differ substantially, is unknown and cannot be addressed from registry data alone. In MDS, *CEBPA* mutations are found in about 1–5% of cases [10], but no study has dedicated analysis to this question: prior large-scale genomic studies incorporated *CEBPA* within multi-gene prognostic panels without dedicated subtype-resolved analysis or transformation-risk quantification [4,5,16]. The aggregate Nres term cannot resolve this issue because it lacks *CEBPA*-specific estimates, mutation subtype effects, or transformation-risk information.

Here, we report the first systematic characterization of *CEBPA* mutation prognostic impact in the large, molecularly annotated IWG 2022 MDS registry (*n* = 2442), examining overall survival, leukemia-free survival, mutation subtype biology, clinical subgroup consistency, the mechanism of the survival effect through competing-risk analysis, and cross-cohort mutation characterization.

## 2. Methods

### 2.1. Study Population and Data Source

**Figure 1:**
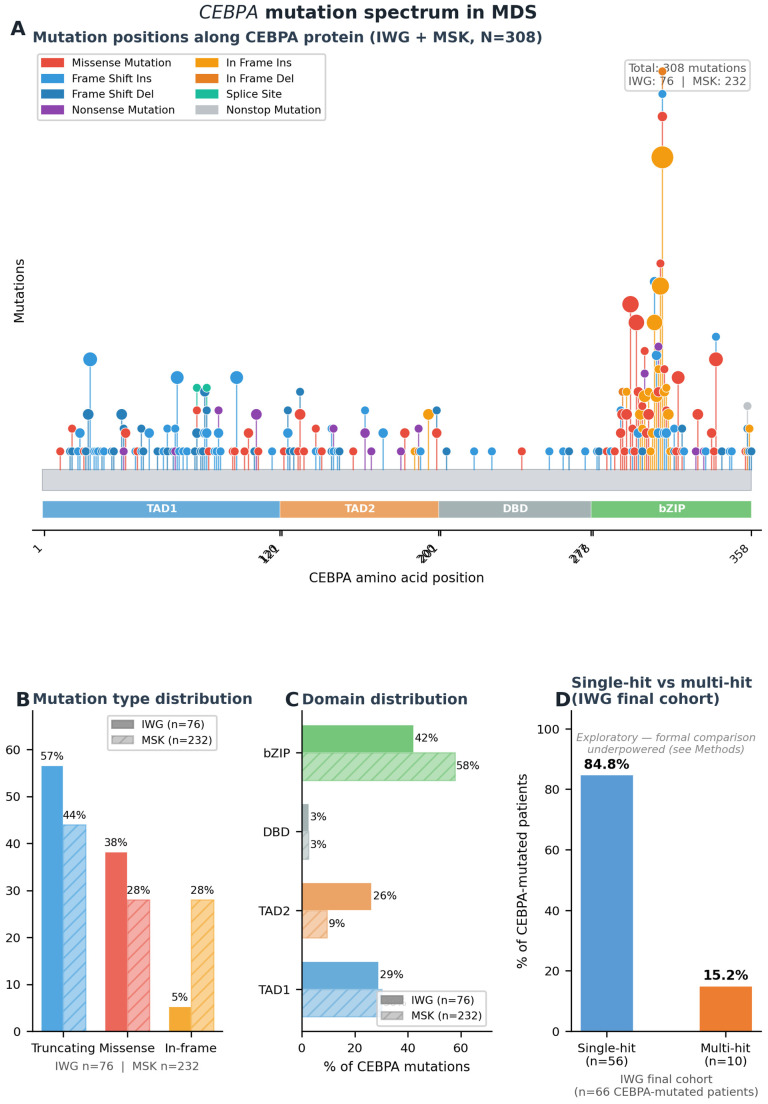
*CEBPA* mutations in MDS are predominantly truncating, with bZIP domain involvement in nearly half of patients and a low multi-hit rate (15.2%). A total of 76 *CEBPA* mutations in 66 IWG final cohort patients (median 1.15 mutations/patient). (**A**) Lollipop plot of mutation positions along the protein (IWG 2022 + MSK 2020 combined; 308 mutations). (**B**) Mutation class distribution (IWG 2022 versus MSK 2020): truncating, missense, and in-frame indels. (**C**) Protein domain distribution (IWG 2022 versus MSK 2020) across TAD1, TAD2, DBD, and bZIP. (**D**) Single-hit (*n* = 56, 84.8%) versus multi-hit (two or more distinct mutations, phasing not confirmed; *n* = 10, 15.2%) in the IWG final cohort. This landscape contrasts with the bZIP in-frame-enriched, high-biallelic context of AML *CEBPA*, underscoring why the AML-derived favorable paradigm cannot be extrapolated to MDS. Note: bZIP, basic leucine zipper; AML, acute myeloid leukemia; DBD, DNA-binding domain; IWG, International Working Group; MDS, myelodysplastic syndromes; TAD, transactivation domain.

The primary analysis cohort was derived from the International Working Group (IWG) 2022 myelodysplastic syndromes registry (Bernard et al. [10]; cBioPortal: mds_iwg_2022) [17,18], a multi-center registry assembled to develop and validate the Molecular IPSS (IPSS-M), containing comprehensive clinical and genomic data with prospectively collected survival endpoints. Patients were included if they had a confirmed MDS diagnosis according to the WHO 2016 classification [1] and available overall survival data; the WHO 2016 classification was used because the registry was constructed on this scheme, and variables required for the WHO 2022 reclassification (fibrosis grade, SF3B1 variant allele frequency threshold, and cellularity) were not captured. Exclusions were applied sequentially: patients classified as having AML (WHO 2016, bone marrow blasts ≥20%; *n* = 175), chronic myelomonocytic leukemia (*n* = 399), and MDS/myeloproliferative neoplasm overlap syndromes (atypical chronic myeloid leukemia, MDS/MPN unclassifiable, and MDS/MPN with ring sideroblasts and thrombocytosis; *n* = 138 combined) were removed; patients with a missing overall survival status or duration (*n* = 132) and those with zero-month survival records (*n* = 37) were subsequently excluded, yielding a final evaluable cohort of 2442 from 3323 starting patients (Supplementary Figure S1). For secondary mutation characterization, we used the MSK-IMPACT 2020 cohort (cBioPortal: mds_mskcc_2020) [19], restricted to MDS-specific Oncotree codes (*n* = 977); this cohort lacks survival endpoints and was used exclusively for cross-cohort mutation comparison. MSK 2020 data were incorporated into the mutation spectrum analysis (Figure 1) alongside IWG 2022 data; per-panel cohort composition is indicated in the figure legend. Both datasets are de-identified and are publicly accessible research resources available via cBioPortal.

### 2.2. Mutation Data and CEBPA Classification

Somatic mutation data were obtained from mutation annotation format (MAF) files via cBioPortal. *CEBPA* mutations were identified by filtering on Hugo_Symbol = ‘*CEBPA*’ and were classified as truncating (frameshift, nonsense, or nonstop), missense, or in-frame (in-frame insertions/deletions). Protein domain assignment used the HGVSp_Short amino-acid position and four functional domains (TAD1, TAD2, DBD, bZIP); domain boundaries are listed in Supplementary Methods. Patients with two or more distinct *CEBPA* mutations were designated as multi-hit (two or more distinct mutations detected; allelic phasing not confirmed); all others were designated as single-hit.

### 2.3. Clinical Variables and Endpoints

The primary endpoint was overall survival (OS; time from sampling to death from any cause), censored at the last follow-up for surviving patients. The secondary endpoint was leukemia-free survival (LFS; time from sampling to AML transformation or death from any cause, whichever occurred first), censored at the last follow-up for patients without either event. Time-zero was set at sampling per the IWG 2022 registry protocol [10]. Baseline clinical covariates considered for analysis included age at diagnosis (continuous), sex, WHO 2016 morphological subtype, MDS etiology (primary versus secondary or therapy-related), IPSS-R score and category [3], complex karyotype status, and cytogenetic risk group. The IPSS-M score [10] was not incorporated into the primary multivariate model because *CEBPA* is one of fifteen genes contributing to the IPSS-M residual mutation term (Nres); adjusting for IPSS-M is therefore circular. An IPSS-M-adjusted sensitivity model is reported in Supplementary Table S1.

### 2.4. Statistical Analysis

Survival distributions were estimated by the Kaplan–Meier method; group differences were evaluated by the two-sided log-rank test. Median survival times are reported with 95% confidence intervals, estimated by the Brookmeyer–Crowley method. Hazard ratios (HR) with 95% confidence intervals were estimated by Cox proportional hazards regression. Two multivariate models were pre-specified: Model A (*CEBPA* + age + sex) and Model B (primary; Model A + continuous IPSS-R score). The proportional hazards assumption was evaluated for each covariate using scaled Schoenfeld residuals; a global test *p*-value below 0.05 was taken as evidence of non-proportionality.

Pre-specified subgroup analyses (seven clinical strata; likelihood ratio interaction tests; Benjamini–Hochberg false discovery rate correction [20] applied to 13 evaluable subgroup hazard ratios), robustness sensitivity analyses (S1–S5), competing-risk analysis (cause-specific Cox [S6]; cumulative incidence by the Aalen–Johansen method with death without AML transformation as the competing event [lifelines 0.30.3, AalenJohansenFitter]; and Fine-Gray subdistribution hazard models for the AML transformation endpoint [R 4.5.2, survival package] [21]), co-mutation–adjusted analyses (S7–S9), co-mutation enrichment testing (Fisher’s exact or chi-squared, Benjamini–Hochberg FDR q < 0.05), and complete-case handling of missing data are detailed in Supplementary Methods. All tests were two-sided (α = 0.05).

### 2.5. Software and Reproducibility

Analyses were performed in Python 3 (pandas, numpy, scipy.stats, statsmodels, lifelines [22], matplotlib, seaborn); the full software stack is detailed in Supplementary Methods.

## 3. Results

### 3.1. Patient Characteristics

After sequential application of the pre-specified exclusion criteria to 3323 IWG 2022 registry patients, the final evaluable cohort comprised 2442 patients (Supplementary Figure S1), of whom 66 (2.7%) carried somatic *CEBPA* mutations, and 2376 (97.3%) were classified as *CEBPA* wild-type. The median follow-up was 45.9 months (3.8 years) for the overall cohort, estimated by the reverse Kaplan–Meier method; the median follow-up was 31.7 months in *CEBPA*-mutated patients and 46.0 months in wild-type patients. The baseline characteristics are summarized in Table 1. The median age was comparable between groups (74.0 versus 72.0 years; *p* = 0.642), as was MDS etiology (*p* = 0.433); the laboratory variables (hemoglobin, platelet count, WBC, and ANC) showed no significant differences (all *p* > 0.10) and are not shown in Table 1, owing to missingness exceeding 15% in this registry (Table 1 footnote). *CEBPA*-mutated patients were more frequently male (72.7% versus 60.0%; *p* = 0.050), had markedly higher bone marrow blast percentages (median 7.5% versus 3.0%; *p* < 0.001), and were enriched in the MDS-EB1 and MDS-EB2 WHO 2016 subtypes (25.4% and 42.9% versus 18.4% and 17.2%, respectively; overall *p* < 0.001). *CEBPA*-mutated patients had higher IPSS-R scores (median 4.5 versus 3.0; *p* < 0.001) and were less likely to carry a complex karyotype (1.5% versus 10.3%; *p* = 0.033). A strictly diploid karyotype was present in 68.3% of *CEBPA*-mutated versus 55.0% of wild-type patients (*p* = 0.048); within the non-complex subgroup, the proportions were 69.5% and 62.0%, respectively (*p* = 0.277; Table 1).

### 3.2. CEBPA Mutation Spectrum

A total of 76 *CEBPA* mutations were identified in 66 patients in the final cohort (with a median of 1.15 mutations per patient). At the mutation class level, frameshift insertions/deletions accounted for 53.9% of the mutations, missense variants for 38.2%, nonsense mutations for 2.6%, and in-frame indels for 5.3% (Figure 1). At the patient level, 36 patients (54.5%) harbored at least one truncating mutation (frameshift or nonsense), 31 (47.0%) had at least one bZIP domain-involving mutation, and 22 (33.3%) had at least one *n*-terminal TAD1 mutation; these categories were not mutually exclusive. Domain assignment showed bZIP (residues 278–358) involvement in 47.0% of patients, TAD1 (residues 1–120) in 33.3%, TAD2 (residues 121–200) in 30.3%, and the DNA-binding domain in 3.0% (Figure 1). A total of 10 patients (15.2%) had two or more distinct *CEBPA* mutations and were designated multi-hit; the remaining 56 (84.8%) were designated single-hit.

### 3.3. Survival Impact of CEBPA Mutations

**Figure 2:**
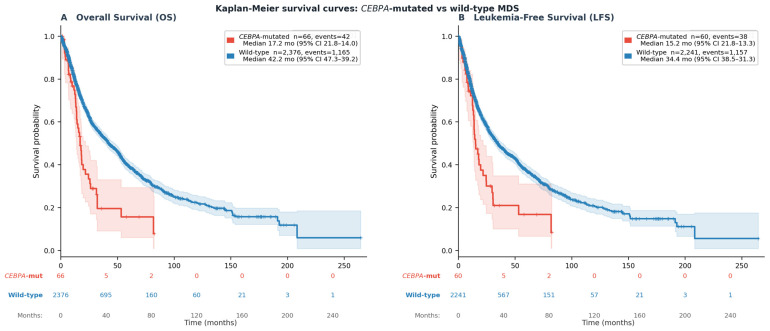
*CEBPA* mutations are associated with markedly inferior overall and leukemia-free survival in MDS, with a more than two-fold unadjusted mortality hazard. Kaplan–Meier curves for *CEBPA*-mutated (*n* = 66; red) and wild-type patients (*n* = 2376; blue); vertical tick marks indicate censored observations. (**A**) OS: median 17.2 months (95% CI 14.0–21.8) versus 42.2 months (95% CI 39.2–47.3); log-rank *p* < 0.001; HR 2.05 (95% CI 1.50–2.79). (**B**) LFS (*n* = 60 *CEBPA*-mutated; *n* = 2241 wild-type): median 15.2 versus 34.4 months; log-rank *p* < 0.001; HR 1.79 (95% CI 1.30–2.48). Number-at-risk tables are shown beneath each panel. Note: CI, confidence interval; HR, hazard ratio; LFS, leukemia-free survival; MDS, myelodysplastic syndromes; OS, overall survival.

**Figure 3:**
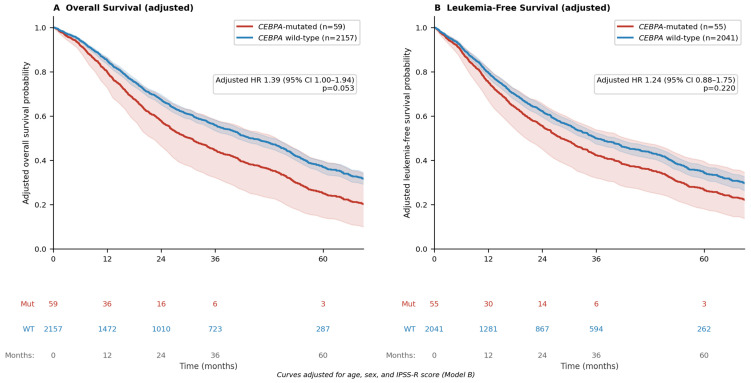
IPSS-R-adjusted Kaplan–Meier survival curves. Cox proportional hazards regression adjusted for age, sex, and continuous IPSS-R score (Model B; population-mean reference). (**A**) OS: *CEBPA*-mutated (*n* = 59) versus wild-type (*n* = 2157); HR 1.39 (95% CI 1.00–1.94; *p* = 0.053). (**B**) LFS: HR 1.24 (95% CI 0.88–1.75; *p* = 0.220). Shaded bands represent 95% CIs. Number-at-risk tables are shown beneath each panel. Note: CI, confidence interval; HR, hazard ratio; IPSS-R, Revised International Prognostic Scoring System; LFS, leukemia-free survival; OS, overall survival.

*CEBPA*-mutated patients had substantially inferior OS compared with wild-type patients (median 17.2 months, 95% CI 14.0–21.8, versus 42.2 months, 95% CI 39.2–47.3; log-rank *p* < 0.001; Figure 2A). A corresponding reduction was observed for leukemia-free survival (median 15.2 months, 95% CI 13.3–21.8, versus 34.4 months, 95% CI 31.3–38.5; *n* = 60 *CEBPA*-mutated patients with LFS data available; log-rank *p* < 0.001; Figure 2B). Upon univariate Cox regression, *CEBPA* mutation status was associated with a more than two-fold increase in the hazard of death (HR 2.05, 95% CI 1.50–2.79; *p* < 0.001) and a significantly elevated LFS hazard (HR 1.79, 95% CI 1.30–2.48; *p* < 0.001) (Table 2). The proportional hazards assumption was satisfied for the *CEBPA* mutation status in both OS models (Schoenfeld *p* = 0.466 for Model A; *p* = 0.321 for Model B); violations in age and IPSS-R score did not affect the direction or validity of the *CEBPA* hazard ratio estimate. In Model A, adjusted for age and sex, the *CEBPA* OS hazard ratio remained substantially elevated (HR 1.93, 95% CI 1.42–2.63; *p* < 0.001), indicating that the survival disadvantage was not explained by the borderline sex imbalance between groups. In primary Model B, which was additionally adjusted for the continuous IPSS-R score, the hazard ratio was attenuated but sustained (OS: HR 1.39, 95% CI 1.00–1.94; *p* = 0.053; *n* = 2216 with complete IPSS-R data; 36 *CEBPA* OS events; LFS: HR 1.24, 95% CI 0.88–1.75; *p* = 0.220) (Table 2, Figure 3). Model C’s results (IPSS-M adjusted) are reported in Supplementary Table S1.

### 3.4. MDS-EB-Restricted Sensitivity Analysis

In an MDS-EB-restricted sensitivity analysis (MDS-EB1 and MDS-EB2 combined; *n* = 870; 562 OS events), *CEBPA* mutation status remained adversely associated with OS upon univariate analysis (HR 1.53, 95% CI 1.07–2.19; *p* = 0.020), and in Model B, adjusted for age, sex, and continuous IPSS-R score (HR 1.48, 95% CI 1.02–2.14; *p* = 0.038), indicating a molecular effect beyond the impact of increased blast burden (Supplementary Table S6, Supplementary Figure S5).

### 3.5. Subgroup Analyses

Pre-specified subgroup analyses were conducted across seven clinical and demographic variables, generating 13 evaluable strata (Supplementary Figure S7, Table 3). The *CEBPA* hazard ratio exceeded 1.0 in all 13 evaluable strata, demonstrating consistent adverse directionality. Nominally significant adverse associations were observed in eight strata on unadjusted testing: an age below the cohort median of 72 years (HR 2.08, 95% CI 1.28–3.38; *p* = 0.003), an age of 72 years or older (HR 1.98, 95% CI 1.33–2.96; *p* < 0.001), female sex (HR 2.21, 95% CI 1.18–4.16; *p* = 0.013), male sex (HR 1.91, 95% CI 1.34–2.72; *p* < 0.001), higher-risk IPSS-R (HR 1.46, 95% CI 1.02–2.09; *p* = 0.037, Benjamini–Hochberg adjusted *p* = 0.060), primary MDS (HR 2.05, 95% CI 1.47–2.85; *p* < 0.001), non-complex karyotype (HR 2.47, 95% CI 1.81–3.37; *p* < 0.001), and good cytogenetic risk (HR 2.59, 95% CI 1.77–3.78; *p* < 0.001) (Table 3). After Benjamini–Hochberg false discovery rate correction was conducted across 13 evaluable strata, the higher-risk IPSS-R association no longer met the conventional threshold (BH-adjusted *p* = 0.060). The lower-risk IPSS-R stratum (HR 1.25, 95% CI 0.52–3.02; *p* = 0.622) and intermediate cytogenetic risk stratum (HR 1.74, 95% CI 0.88–3.43; *p* = 0.110) were designated exploratory, owing to fewer than ten *CEBPA* events. No significant interaction was detected between *CEBPA* status and any subgroup variable (all *p*_interactions were 0.552–0.924), confirming uniform directionality across all clinical contexts.

### 3.6. CEBPA Mutation Subtype Analysis

Power screening confirmed two powered subtype comparisons (Supplementary Table S2). Patients with truncating *CEBPA* mutations (*n* = 36) had significantly inferior OS rates compared with non-truncating patients (*n* = 30; HR 2.21, 95% CI 1.12–4.37; *p* = 0.023; Supplementary Figure S2). The bZIP-domain mutations conferred no detectable survival differences (*n* = 31 versus *n* = 35; HR 1.25, 95% CI 0.68–2.31; *p* = 0.470; Supplementary Figure S2). The multi-hit subtype (*n* = 10, 15.2%) was treated as descriptive, given insufficient power; the median OS rate was 18.4 versus 17.2 months in single-hit patients (Supplementary Figure S3). We additionally applied the narrow bZIP in-frame insertion/deletion definition that has emerged as the AML-favorable subset in recent reports [23]; only two patients in the cohort met this strict definition (p.N293del and p.N356_C357del), contributing one OS and one LFS event each. Because the events-per-predictor ratio was far below the pre-specified threshold of ten, the bZIP in-frame indel comparison is reported as descriptive only (OS HR 0.43, 95% CI 0.06–3.18, *p* = 0.412; LFS HR 0.44, 95% CI 0.06–3.24, *p* = 0.422), and the AML-derived favorable signal could not be formally evaluated in this MDS cohort.

### 3.7. Co-Mutation Landscape

**Figure 4:**
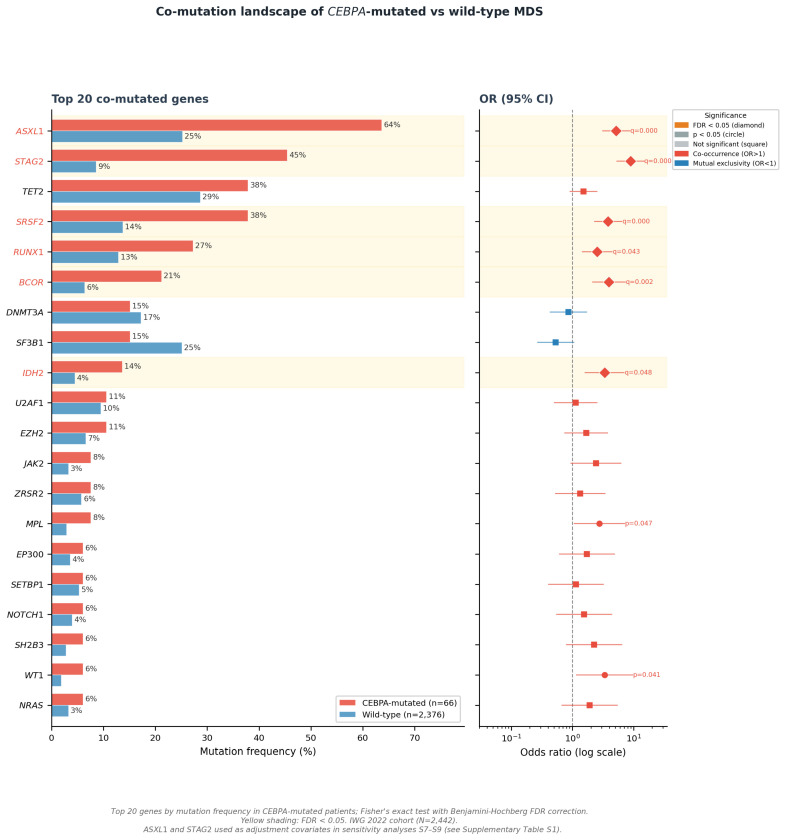
*STAG2* and *ASXL1* are markedly co-enriched in *CEBPA*-mutated MDS patients, defining a co-mutational context of established adverse prognostic significance. Mutation frequencies in *CEBPA*-mutated (*n* = 66; red) and wild-type patients (*n* = 2376; blue). Six genes reached Benjamini–Hochberg FDR significance (q < 0.05): *STAG2* (OR 8.87; q = 1.02 × 10^−12^), *ASXL1* (OR 5.18; q = 4.87 × 10^−9^), *BCOR* (OR 3.94; q = 1.90 × 10^−3^), *SRSF2* (OR 3.82; q = 4.25 × 10^−5^), *IDH2* (OR 3.35; q = 0.048), and *RUNX1* (OR 2.53; q = 0.043). *TET2* was not enriched (q = 0.391). *TP53* was nominally depleted in *CEBPA*-mutated patients (OR 0.21; nominal *p* = 0.013; q = 0.142). Note: FDR, false discovery rate; MDS, myelodysplastic syndromes; OR, odds ratio.

Six genes were FDR-significantly co-enriched in *CEBPA*-mutated patients relative to wild-type patients (Figure 4, Supplementary Table S4). *CEBPA*-mutated patients harbored a higher overall co-mutation burden than wild-type patients (median 5.0 non-*CEBPA* mutated genes, IQR 4.0–6.8, versus median 4.0, IQR 2.0–5.0; Wilcoxon *p* < 0.001). The strongest co-occurrence by odds ratio was observed for *STAG2* (45.5% versus 8.6%; OR 8.87; q = 1.02 × 10^−12^), followed by *ASXL1* (63.6% versus 25.3%; OR 5.18; q = 4.87 × 10^−9^), *BCOR* (21.2% versus 6.4%; OR 3.94; q = 1.90 × 10^−3^), *SRSF2* (37.9% versus 13.8%; OR 3.82; q = 4.25 × 10^−5^), *IDH2* (13.6% versus 4.5%; OR 3.35; q = 0.048), and *RUNX1* (27.3% versus 12.9%; OR 2.53; q = 0.043). *TP53* showed a nominally lower frequency in *CEBPA*-mutated patients (3.0% versus 13.0%; OR 0.21; *p* = 0.013), which is consistent with the predominantly diploid, cytogenetically stable background in this subgroup. These co-occurrence patterns raise the possibility that the observed survival association reflects *CEBPA*-mutated patients’ membership in a broader adverse molecular cluster (frequent co-mutation with *STAG2*, *ASXL1*, and *SRSF2*) rather than as an effect that is independent of the co-mutation context.

### 3.8. Sensitivity Analyses

**Figure 5:**
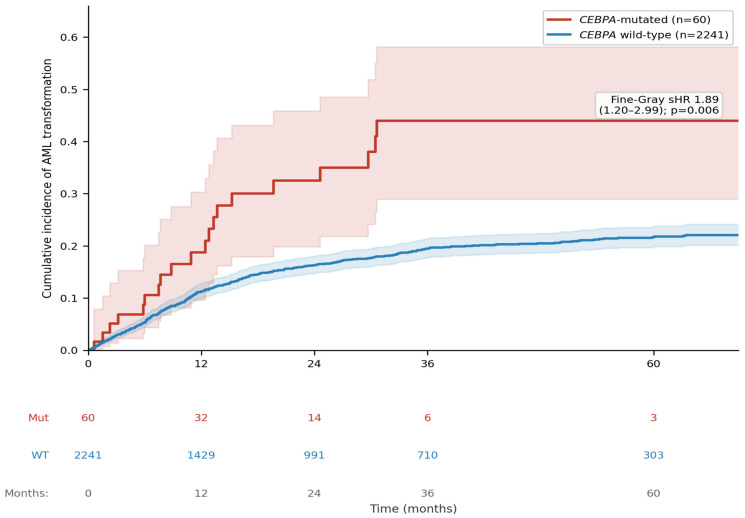
Aalen–Johansen cumulative incidence of AML transformation by *CEBPA* mutation status. AML transformation cumulative incidence at 36 months: 44.0% (95% CI 28.9–58.1%) in *CEBPA*-mutated patients (*n* = 60) versus 19.5% (95% CI 17.7–21.3%) in wild-type patients (*n* = 2241). Fine-Gray subdistribution hazard ratio for AML transformation: 1.89 (95% CI 1.20–2.99; *p* = 0.006), adjusted for age, sex, and IPSS-R. Shaded bands represent 95% CIs. Competing events include death without AML transformation. Number-at-risk counts are shown beneath. Note: AML, acute myeloid leukemia; CI, confidence interval.

The primary multivariate finding was robust across sensitivity analyses S1–S5 (Supplementary Table S1). The *CEBPA* Model B hazard ratio ranged from 1.38 to 1.53; the 95% CI lower bound crossed 1.0 in S4 (HR 1.38, 95% CI 0.98–1.94; *p* = 0.07) but not in S1 (HR 1.43, 95% CI 1.02–1.99; *p* = 0.037), S2 (HR 1.42, 95% CI 1.00–2.01; *p* = 0.049), or S5 (HR 1.53, 95% CI 1.09–2.14; *p* = 0.014). The landmark analysis (S5), initiated at three months to condition on early survival, yielded the largest hazard ratio, arguing against guarantee-time bias. In the competing-risk analysis (S6), in which AML transformation was the event of interest and death without transformation was the competing event, the cause-specific Cox hazard ratio for *CEBPA* status in the death-without-transformation model was 0.97 (95% CI 0.587–1.588; *p* = 0.890). This indicates that *CEBPA*-mutated patients who remain in MDS without transforming do not die faster than wild-type patients; of the 36 primary *CEBPA* OS events, 20 were attributed to AML transformation, confirming leukemic evolution as the dominant mortality pathway (Supplementary Table S1). Overall, AML transformation occurred in 33.3% of evaluable *CEBPA*-mutated patients (crude proportion 20/60) versus 18.0% of wild-type patients (404/2241). On a Fine-Gray subdistribution hazard model adjusted for age, sex, and IPSS-R, the subdistribution hazard of AML transformation was significantly elevated (subdistribution HR 1.89, 95% CI 1.20–2.99; *p* = 0.006). The Aalen–Johansen cumulative incidence of AML transformation at 36 months was 44.0% (95% CI 28.9–58.1%) in *CEBPA*-mutated versus 19.5% (95% CI 17.7–21.3%) in wild-type patients (Figure 5; Supplementary Table S1); the median time to transformation was similar between groups (11.7 months, IQR 6.0–16.4, versus 10.6 months, IQR 5.4–20.8). Within the *CEBPA*-mutated cohort, the transformation rates did not differ significantly by mutation subtype: truncating versus non-truncating (42.4% versus 22.2%; *p* = 0.168), bZIP-involving versus non-bZIP (41.4% versus 25.8%; *p* = 0.275), or multi-hit versus single-hit (50.0% versus 30.0%; *p* = 0.278), indicating that an elevated leukemic transformation risk is a class-level rather than a subtype-specific property of *CEBPA* mutation in MDS. The univariate Cox results for all 17 variables are presented in Supplementary Table S5.

### 3.9. Cross-Cohort Mutation Characterization

In the MSK-IMPACT 2020 cohort filtered to MDS-specific Oncotree codes (*n* = 977), *CEBPA* mutations were identified in 14 patients (1.4%), compared with 2.7% in the IWG cohort (chi-square *p* = 0.026) (Supplementary Table S3). The multi-hit proportion (two or more distinct mutations) was similar between cohorts (IWG 15.2% versus MSK 19.2%; *p* = 0.465), representing the most directly comparable biological feature between datasets. The mutation type distribution differed significantly (chi-square *p* < 0.001): the MSK cohort showed higher frequencies of in-frame insertions (28.0% versus 5.3% in IWG), which is attributable to an admixture of AML cases for which bZIP in-frame insertions are characteristic. Survival data were unavailable in the MSK cohort; the primary prognostic findings could not be externally validated (Supplementary Table S3). In the IWG cohort, variant allele frequencies (VAFs) were available for all 76 *CEBPA* mutations; the median VAF was 0.231 (IQR 0.098–0.454), and 34 of 76 mutations (44.7%) fell below the conventional 20% subclonal threshold, indicating that a substantial proportion of *CEBPA* hits in MDS arise at subclonal allele frequencies (Supplementary Figure S6).

## 4. Discussion

In this large, molecularly annotated MDS cohort, *CEBPA* mutations were present in 2.7% of patients and were associated with markedly inferior OS rates: median 17.2 versus 42.2 months in wild-type patients, corresponding to a more than two-fold unadjusted mortality hazard (HR 2.05, 95% CI 1.50–2.79; *p* < 0.001). After an adjustment for age, sex, and IPSS-R score in Model B, *CEBPA* retained a borderline adverse hazard ratio (HR 1.39, 95% CI 1.00–1.94; *p* = 0.053; Figure 3); the direction of effect was consistent across all 13 evaluable subgroups, with no interaction reaching significance. Because the bone marrow blast percentage (an IPSS-R component) is markedly elevated in *CEBPA*-mutated patients at baseline (median 7.5% versus 3.0%; *p* < 0.001), Model B partially absorbs the *CEBPA* effect through this blast-mediated pathway and should be viewed as the residual association beyond age, sex, and IPSS-R. Competing-risk analysis suggests that the excess mortality may be mediated through AML transformation (cause-specific HR 0.97; *p* = 0.890 for death without transformation). Among mutation subtypes, truncating mutations were associated with the adverse signal (HR 2.21; *p* = 0.023), while bZIP domain mutations were not associated with a significant survival advantage (HR 1.25; *p* = 0.470), diverging from the established AML *CEBPA* paradigm.

Prior genomic studies have consistently identified recurrently mutated genes as independent prognostic determinants beyond the IPSS-R [4,5], but *CEBPA* has not been separately characterized; it has been incorporated as one of fifteen genes in the IPSS-M Nres term without independent phenotypic characterization [10]. To our knowledge, with 66 *CEBPA*-mutated patients drawn from a 2442-patient registry, this represents the largest dedicated prognostic analysis of *CEBPA*-mutated MDS with linked survival outcomes and the first systematic description of *CEBPA*’s clinical and prognostic associations in MDS. Prior large-scale genomic surveys (439, 738, and 944 patients in the series of Bejar et al. [4], Papaemmanuil et al. [5], and Haferlach et al. [16], respectively) reported a prevalence of approximately 2% without dedicated analysis of the mutated subgroup. Our observed prevalence of 2.7% is consistent with published MDS cohorts [4,10]. The co-mutation landscape is dominated by genes of established adverse prognostic significance: *STAG2* (OR 8.87) and *ASXL1* (OR 5.18) were the most strongly enriched [5,24], and both were independently associated with inferior outcomes in MDS, suggesting that *CEBPA* mutations tend to arise within a molecularly aggressive clonal context. Co-mutation-adjusted sensitivity analyses (S7–S9; adjusting for *ASXL1*, *STAG2*, or both) attenuated the *CEBPA* hazard ratio in a stepwise fashion to a floor of HR 1.11 (95% CI 0.79–1.57; *p* = 0.54), while preserving the adverse direction throughout (Supplementary Table S1; Supplementary Figure S4). This pattern is consistent with the *CEBPA* mutation status functioning partly as a surrogate marker for an adverse co-mutation milieu rather than as an independent effector of mortality; the residual adverse direction (HR 1.11) across all adjusted models, however, suggests a contribution beyond co-mutation burden alone. The near-complete depletion of *TP53* co-mutation (3.0% versus 13.0%; *p* = 0.013) and low complex karyotype frequency (1.5%) further confirm that *CEBPA*-mutated MDS represents a cytogenetically stable, diploid disease context that is distinct from the *TP53*/complex karyotype MDS subtype.

The phenotype of *CEBPA*-mutated MDS differs from that of *CEBPA*-mutated AML across several axes. Patients in this cohort were considerably older (median 74.0 years) than the reported AML bZIP in-frame indel cohorts (42 to 47 years in Georgi et al. [23] and 49.6 years in the PETHEMA registry [25]). Multi-hit involvement (two or more distinct mutations, without phasing confirmation) was substantially less frequent in MDS (15.2%) than the biallelic rate of approximately 50% reported in pooled AML *CEBPA*-mutated series [23]. The co-mutation profile also diverges: MDS *CEBPA*-mutated patients were enriched for *ASXL1* (63.6%), *STAG2* (45.5%), and *SRSF2* (37.9%), all uncommon in AML bZIP in-frame indel cohorts, which are instead enriched for *GATA2* and *WT1* with near-absent *NPM1* co-mutation [23,25]; in the present MDS cohort, *GATA2* was not observed, and *NPM1* was present in only 3.0% of *CEBPA*-mutated patients. The *CEBPA*-mutated MDS profile thus resembles the AML *CEBPA-other* category (older patients, splicing and chromatin co-mutations) more closely than the AML bZIP in-frame indel subset. These cross-disease comparisons are observational and draw on different registries with distinct selection criteria and molecular panels; direct statistical comparison was not performed.

The data presented in this manuscript suggest that the *CEBPA* prognostic signal does not support the presence of an AML-favorable risk in MDS. In the present cohort, bZIP-domain mutations (which define ELN 2022 Favorable risk in AML) conferred no survival benefit (HR 1.25; *p* = 0.470), and multi-hit patients (*n* = 10; median OS 18.4 months) fared no better than single-hit patients. Two mechanisms account for this divergence. First, the chemosensitivity conferred by bZIP mutations requires intensive anthracycline-based induction, which is rarely used in MDS, where hypomethylating agents predominate [26]. Recent AML data report high complete response rates with venetoclax-plus-hypomethylating agent combinations [27], and *CEBPA*-mutated AML may share this sensitivity [28], although durability appears limited, and whether this extends to MDS with *CEBPA* mutations remains untested. Second, *CEBPA* mutations in MDS occur within a co-mutational milieu marked by frequent *ASXL1*, *STAG2*, and *SRSF2*, strikingly different from the mutation-sparse context of biallelic AML *CEBPA*, and this adverse co-mutation background may attenuate or negate any differentiation competence conferred by the bZIP mutation in this disease context [14]. The mutation spectrum (Figure 1) contrasts with the bZIP in-frame-enriched high-biallelic context of AML *CEBPA* and underscores why the AML-derived favorable paradigm cannot be extrapolated to MDS.

The mechanism by which *CEBPA* mutations confer adverse prognosis in MDS appears to operate primarily through the promotion of leukemic transformation rather than direct acceleration of MDS-related mortality. The null competing-risk result (S6: cause-specific HR 0.97, *p* = 0.890) indicates that *CEBPA*-mutated patients who remain in MDS without transforming do not die faster than wild-type patients. Of the 36 primary *CEBPA* OS events, 20 occurred as AML transformation, corresponding to a cumulative transformation rate of 33.3% across evaluable *CEBPA*-mutated patients (20/60 with LFS data), which is nearly double the 18.0% rate in wild-type patients (404/2241). C/EBPα, encoded by *CEBPA*, is a master transcription factor required for granulocytic commitment [11]; truncating mutations that ablate the N-terminal TAD1 transactivation domain disrupt this differentiation function, creating a maturation block that predisposes to blast accumulation and leukemic progression, which is consistent with the higher bone marrow blast percentages at baseline (median 7.5% versus 3.0%; *p* < 0.001). Truncating mutations were associated with a numerically higher transformation rate than non-truncating mutations (42.4% versus 22.2%), with a parallel gradient observed across bZIP-involving versus non-bZIP mutations (41.4% versus 25.8%); neither subtype difference reached statistical significance in the current cohort (*p* = 0.168 and *p* = 0.275, respectively), but the directional concordance with the independent truncating mutation survival signal (HR 2.21, *p* = 0.023) strengthens the mechanistic interpretation.

From a clinical standpoint, the most actionable implication is that the AML-derived bZIP-favorable paradigm should not be applied to *CEBPA*-mutated MDS patients when discussing prognosis or treatment: neither bZIP involvement nor multi-hit status conferred any survival advantage in this cohort. The IPSS-R already captures elements of *CEBPA* biology indirectly through the blast percentage and cytogenetic risk, but not through molecular annotation; a *CEBPA*-mutated patient in the IPSS-R lower-risk category may carry a higher transformation risk than their IPSS-R score indicates; the hazard ratio point estimate in that stratum remains adverse (HR 1.25). The significant co-enrichment of *IDH2* (13.6%; OR 3.35; q = 0.048) identifies a potentially targetable subgroup, warranting a dedicated investigation of *IDH2* inhibitor therapy [29] in *CEBPA*-mutated MDS. Treatment decisions should not be guided by AML-derived chemosensitivity assumptions; current evidence supports a prospective study design to evaluate whether molecular risk stratification in this subgroup has clinical utility.

This study has several limitations. With 66 *CEBPA*-mutated patients and 36 OS events in Model B, the multivariate hazard ratio of 1.39 (95% CI 1.00–1.94) sits at the boundary of statistical significance and warrants confirmation in larger datasets. The primary model was adjusted for established clinical factors but not for molecular co-mutations; pre-specified co-mutation-adjusted analyses (S7–S9) attenuated the *CEBPA* hazard ratio in a stepwise fashion to a floor of HR 1.11 while preserving an adverse direction, indicating that the true effect lies in the range HR 1.11–2.05, depending on the adjustment applied [4,24]. The IWG 2022 registry does not include treatment data, precluding analyses of treatment effects, transplantation access, or the bZIP chemosensitivity hypothesis. External survival validation was not possible: the MSK 2020 cohort, the largest independent *CEBPA* MDS dataset, lacks survival endpoints. Age and the IPSS-R score violated the proportional hazards assumption (Schoenfeld *p* < 0.001), although the *CEBPA* mutation status itself satisfied the assumption. IPSS-R data were missing in 7 of 66 *CEBPA*-mutated patients (10.6%), and LFS data were missing in 6 of 66 (9.1%) patients. Within the *CEBPA*-mutated subset, those with missing IPSS-R data had markedly shorter median OS rates than those with complete data (6.97 versus 15.19 months; *p* = 0.041), consistent with informative missingness that biases the Model B HR toward the null. A dedicated subgroup comparison of isolated (sole) *CEBPA* mutations versus co-mutated cases was not pre-specified and was not feasible because all *CEBPA*-mutated patients carried at least one additional somatic mutation, leaving no isolated cases; the sensitivity analyses S7–S9 partially address this by adjusting for the most strongly enriched co-mutations. The broad VAF distribution (44.7% of mutations below the 20% subclonal threshold) suggests that a proportion of *CEBPA* mutations may arise as subclonal or early predisposing events; longitudinal studies are needed to determine their clonal hierarchy and pre-leukemic role [30]. Finally, this analysis is retrospective and observational; the associations are prognostic and do not permit causal inference.

## 5. Conclusions

*CEBPA* mutations identify a high-risk MDS subgroup with markedly inferior survival, a signal driven primarily by truncating loss-of-function mutations and mediated through leukemic transformation, partly reflecting an adverse co-mutation milieu. The AML-derived bZIP-favorable paradigm does not translate to MDS. *CEBPA* mutation status merits a prospective study to determine its clinical utility for risk stratification in MDS.

## Figures and Tables

**Table 1:** Baseline clinical and molecular characteristics of *CEBPA*-mutated and *CEBPA* wild-type patients in the IWG 2022 MDS cohort (*n* = 2442).

Characteristic	All Patients	*CEBPA*-Mutated	Wild-Type	*p*-Value
Age, median (IQR)	72.0 (63.8–78.0)	74.0 (67.0–77.0)	72.0 (63.0–78.0)	0.642
Sex	*n* = 2442	*n* = 66	*n* = 2376	0.050
Male	1473 (60.3%)	48 (72.7%)	1425 (60.0%)	
Female	969 (39.7%)	18 (27.3%)	951 (40.0%)	
WHO 2016 classification	*n* = 2388	*n* = 63	*n* = 2325	<0.001
MDS-MLD	613 (25.7%)	12 (19.0%)	601 (25.8%)	
MDS-EB1	444 (18.6%)	16 (25.4%)	428 (18.4%)	
MDS-EB2	426 (17.8%)	27 (42.9%)	399 (17.2%)	
MDS-RS-SLD	247 (10.3%)	3 (4.8%)	244 (10.5%)	
MDS-RS-MLD	205 (8.6%)	1 (1.6%)	204 (8.8%)	
MDS-SLD	179 (7.5%)	2 (3.2%)	177 (7.6%)	
MDS-del5q	138 (5.8%)	0 (0.0%)	138 (5.9%)	
MDS-U	82 (3.4%)	1 (1.6%)	81 (3.5%)	
MDS-SLD/MLD	47 (2.0%)	0 (0.0%)	47 (2.0%)	
other	4 (0.2%)	0 (0.0%)	4 (0.2%)	
MDS-RS-SLD/MLD	3 (0.1%)	1 (1.6%)	2 (0.1%)	
Missing	54 (2.2%)			
MDS type	*n* = 2442	*n* = 66	*n* = 2376	0.433
primary	2138 (87.6%)	61 (92.4%)	2077 (87.4%)	
Secondary/therapy-related	217 (8.9%)	3 (4.5%)	214 (9.0%)	
Unknown	87 (3.6%)	2 (3.0%)	85 (3.6%)	
BM blast %, median (IQR)	3.0 (1.0–7.0)	7.5 (3.5–11.5)	3.0 (1.0–7.0)	<0.001
PB blast %, median (IQR)	0.0 (0.0–0.0)	0.0 (0.0–0.0)	0.0 (0.0–0.0)	0.003
IPSS-R category	*n* = 2302	*n* = 60	*n* = 2242	<0.001
Very Low	371 (16.1%)	6 (10.0%)	365 (16.3%)	
Low	884 (38.4%)	9 (15.0%)	875 (39.0%)	
Int	475 (20.6%)	24 (40.0%)	451 (20.1%)	
High	320 (13.9%)	15 (25.0%)	305 (13.6%)	
Very High	252 (10.9%)	6 (10.0%)	246 (11.0%)	
Missing	140 (5.7%)			
IPSS-R score, median (IQR)	3.0 (2.0–4.5)	4.5 (3.5–5.5)	3.0 (2.0–4.5)	<0.001
IPSS-M category	*n* = 2231	*n* = 57	*n* = 2174	<0.001
Very Low	278 (12.5%)	2 (3.5%)	276 (12.7%)	
Low	716 (32.1%)	8 (14.0%)	708 (32.6%)	
Moderate-Low	252 (11.3%)	4 (7.0%)	248 (11.4%)	
Moderate-High	243 (10.9%)	4 (7.0%)	239 (11.0%)	
High	318 (14.3%)	15 (26.3%)	303 (13.9%)	
Very High	424 (19.0%)	24 (42.1%)	400 (18.4%)	
Missing	211 (8.6%)			
IPSS-M score, median (IQR)	−0.3 (−1.1–1.0)	1.4 (0.1–1.9)	−0.3 (−1.1–1.0)	<0.001
Cytogenetic risk (IPSS-R)	*n* = 2334	*n* = 62	*n* = 2272	0.017
Very Good	95 (4.1%)	1 (1.6%)	94 (4.1%)	
Good	1634 (70.0%)	46 (74.2%)	1588 (69.9%)	
Int	302 (12.9%)	14 (22.6%)	288 (12.7%)	
Poor	109 (4.7%)	0 (0.0%)	109 (4.8%)	
Very Poor	194 (8.3%)	1 (1.6%)	193 (8.5%)	
Missing	108 (4.4%)			
Complex karyotype	*n* = 2442	*n* = 66	*n* = 2376	0.033
Complex	246 (10.1%)	1 (1.5%)	245 (10.3%)	
Non-complex	2196 (89.9%)	65 (98.5%)	2131 (89.7%)	
Normal karyotype (within non-complex)	1200 (62.3%)	41 (69.5%)	1159 (62.0%)	0.277

Data are the medians (interquartile range) for continuous variables and *n* (%) for categorical variables. *p*-values: Wilcoxon rank-sum test for continuous variables; chi-squared or Fisher’s exact tests for categorical variables, as appropriate. IPSS-R data were missing for 226 patients (9.3%; *CEBPA*-mutated *n* = 7, wild-type *n* = 219); leukemia-free survival endpoints were missing for 141 patients (5.8%; *CEBPA*-mutated *n* = 6, wild-type *n* = 135). Standardized differences (SMD): age 0.06, sex 0.27, WHO 2016 subtype 0.88, MDS type 0.18, BM blast % 0.80, PB blast % 0.50, IPSS-R category 0.71, IPSS-R score 0.37, IPSS-M category 0.80, IPSS-M score 0.65, cytogenetic risk 0.54, and complex karyotype 0.38; computed as Cohen’s d for continuous variables and the Yang–Dalton standardized difference for categorical variables. Laboratory variables (hemoglobin, platelet count, WBC, and ANC) were excluded from Table 1, owing to missingness exceeding 15% in this registry cohort; these variables showed no significant differences between groups (all *p*  > 0.10). Note: IQR, interquartile range; IPSS-R, Revised International Prognostic Scoring System; MDS, myelodysplastic syndromes; MDS-EB, MDS with excess blasts; MDS-MLD, MDS with multilineage dysplasia; MDS-RS, MDS with ring sideroblasts; MDS-SLD, MDS with single lineage dysplasia; MDS-U, MDS unclassifiable; WHO, World Health Organization.

**Table 2:** Univariate and multivariate Cox proportional hazard regressions for overall survival and leukemia-free survival.

Variable	*n*	Events	HR	95% CI	*p*-Value
***Overall Survival, Univariate***					
*CEBPA* mutation (mutated vs. WT)	2442	1207	2.05	1.50–2.79	<0.001
Age (per year)	2440	1206	1.02	1.02–1.03	<0.001
Sex (male vs. female)	2442	1207	1.30	1.15–1.46	<0.001
BM blast % (per unit)	2373	1166	1.08	1.07–1.10	<0.001
IPSS-R score (per unit)	2217	1074	1.41	1.37–1.45	<0.001
Complex karyotype (vs. non-complex)	2442	1207	4.24	3.62–4.96	<0.001
MDS type (s/t-MDS vs. primary)	2355	1158	1.64	1.37–1.96	<0.001
***Leukemia-Free Survival, Univariate***					
*CEBPA* mutation (mutated vs. WT)	2301	1195	1.79	1.30–2.48	<0.001
Age (per year)	2300	1194	1.02	1.01–1.02	<0.001
Sex (male vs. female)	2301	1195	1.32	1.17–1.48	<0.001
BM blast % (per unit)	2246	1160	1.10	1.09–1.11	<0.001
IPSS-R score (per unit)	2097	1072	1.40	1.36–1.44	<0.001
Complex karyotype (vs. non-complex)	2301	1195	3.90	3.32–4.57	<0.001
MDS type (s/t-MDS vs. primary)	2231	1150	1.60	1.34–1.91	<0.001
***Overall Survival, Multivariate***					
**Model A: *CEBPA* + Age + Sex**	2440	1206			
*CEBPA* mutation (mutated vs. WT)			1.93	1.42–2.63	<0.001
Age (per year)			1.02	1.02–1.03	<0.001
Sex (male vs. female)			1.27	1.12–1.42	<0.001
**Model B: *CEBPA* + Age + Sex + IPSS-R score [PRIMARY]**	2216	1073			
*CEBPA* mutation (mutated vs. WT)			1.39	1.00–1.94	0.053
Age (per year)			1.03	1.03–1.04	<0.001
Sex (male vs. female)			1.19	1.05–1.35	0.006
IPSS-R score (per unit)			1.46	1.42–1.50	<0.001
***Leukemia-Free Survival, Multivariate***					
**Model A: *CEBPA* + Age + Sex**	2300	1194			
*CEBPA* mutation (mutated vs. WT)			1.68	1.21–2.32	0.002
Age (per year)			1.02	1.01–1.02	<0.001
Sex (male vs. female)			1.28	1.14–1.44	<0.001
**Model B: *CEBPA* + Age + Sex + IPSS-R score [PRIMARY]**	2096	1071			
*CEBPA* mutation (mutated vs. WT)			1.24	0.88–1.75	0.220
Age (per year)			1.03	1.02–1.04	<0.001
Sex (male vs. female)			1.23	1.09–1.40	0.001
IPSS-R score (per unit)			1.44	1.40–1.49	<0.001

Model A: adjusted for *CEBPA* mutation status, age (continuous), and sex. Model B (primary model): additionally adjusted for IPSS-R score (continuous); restricted to patients with complete IPSS-R data (*n* = 2216; *CEBPA*-mutated *n* = 59, 36 events). The IPSS-M was withheld from the primary model as *CEBPA* is encoded in the IPSS-M residual mutation term (Nres); IPSS-M-adjusted results are presented in Supplementary Table S1. The proportional hazards assumption was evaluated by scaled Schoenfeld residuals; *CEBPA* mutation status satisfied the assumption in all models (Schoenfeld *p* = 0.321–0.466 for OS; *p* = 0.335–0.362 for LFS). Yellow shading highlights the primary Model B results (CEBPA mutation status; OS HR 1.39, 95% CI 1.00–1.94; *p* = 0.053). Note: CI, confidence interval; HR, hazard ratio; IPSS-M, Molecular International Prognostic Scoring System; IPSS-R, Revised International Prognostic Scoring System; LFS, leukemia-free survival; OS, overall survival.

**Table 3:** Subgroup analyses: stratum-specific hazard ratios and interaction test results for the association between *CEBPA* mutation status and overall survival (*n* = 2442).

Subgroup	*n* (Total)	*n CEBPA*	*CEBPA* Events	HR (OS)	95% CI	*p*-Value	*p* Interaction
Age							
Age < 72 yr	1200	30	17	2.08	1.28–3.38	0.003	0.643
Age ≥ 72 yr	1240	36	25	1.98	1.33–2.96	<0.001	0.643
Sex							
Female	969	18	10	2.21	1.18–4.16	0.013	0.725
Male	1473	48	32	1.91	1.34–2.72	<0.001	0.725
IPSS-R risk							
IPSS-R: Lower risk (VL + L) *	1255	15	5	1.25	0.52–3.02	0.622	0.552
IPSS-R: Higher risk (Int + H + VH)	1047	45	32	1.46	1.02–2.09	0.037	0.552
WHO 2016 subtype							
WHO: MDS-EB1	444	16	12	1.61	0.90–2.88	0.108	0.924
WHO: MDS-EB2	426	27	20	1.38	0.87–2.17	0.172	0.924
WHO: MDS-MLD *	613	12	6	1.68	0.75–3.77	0.211	0.924
MDS type							
primary	2138	61	37	2.05	1.47–2.85	<0.001	0.740
s_t_MDS	217	3	3		NaN–NaN	N/A (*n* = 3)	0.740
Complex karyotype							
Karyotype: complex	246	1	0		NaN–NaN	N/A (*n* = 1)	0.634
Karyotype: non-complex	2196	65	42	2.47	1.81–3.37	<0.001	0.634
Cytogenetics (IPSS-R)							
CYTO: Good	1634	46	28	2.59	1.77–3.78	<0.001	0.602
CYTO: Int *	302	14	9	1.74	0.88–3.43	0.110	0.602
CYTO: Poor/Very Poor	303	1	0		NaN–NaN	N/A (*n* = 1)	0.602

Hazard ratios with 95% CIs are from stratum-specific Cox proportional hazards models with *CEBPA* mutation status used as the sole covariate (unadjusted within stratum). Interaction *p*-values are from likelihood ratio tests comparing models with and without a multiplicative *CEBPA* × subgroup interaction term. Benjamini–Hochberg false discovery rate correction was applied across the 13 evaluable strata; no stratum remained significant at q < 0.05 (the only nominally significant result, higher-risk IPSS-R *p* = 0.037, had an adjusted *p* of 0.060). Interaction testing also served as a multiplicity control. * Exploratory: fewer than 10 *CEBPA*-related OS events. Note: CI, confidence interval; HR, hazard ratio; IPSS-R, Revised International Prognostic Scoring System; N/A, not applicable; OS, overall survival; WHO, World Health Organization.

## Data Availability

The IWG 2022 (cBioPortal study ‘mds_iwg_2022’) and MSK-IMPACT 2020 (‘mds_mskcc_2020’) datasets analyzed in this study are publicly accessible at https://www.cbioportal.org/ (accessed on 27 June 2026).

## Supplementary Materials

The following supporting information can be downloaded at: https://www.mdpi.com/article/10.3390/cancers18132135/s1, Supplementary Table S1. Pre-specified sensitivity analyses: *CEBPA* Model B hazard ratio across nine analytic scenarios. Supplementary Table S2. *CEBPA* mutation subtype power analysis and descriptive characterisation. Supplementary Table S3. Cross-cohort *CEBPA* mutation characterisation: IWG 2022 versus MSK-IMPACT 2020. Supplementary Table S4. Co-mutation enrichment analysis: gene-level co-occurrence in *CEBPA*-mutated versus wild-type MDS patients (IWG 2022 cohort, *n* = 2442). Supplementary Table S5. Univariate Cox proportional hazards regression for all tested variables - overall survival and leukaemia-free survival. Supplementary Table S6. Cox proportional hazards regression restricted to the MDS-EB subcohort (MDS-EB1 + MDS-EB2; *n* = 870; OS events = 562). Supplementary Figure S1. *CEBPA* mutations were identified in 66 of 2442 evaluable MDS patients (2.7%) after sequential pre-specified diagnostic exclusions. Supplementary Figure S2. Truncating *CEBPA* mutations drive the adverse survival signal in MDS; bZIP domain mutations confer no detectable survival effect. Supplementary Figure S3. Multi-hit *CEBPA* mutations do not confer survival benefit in MDS - descriptive comparison, no formal test. Supplementary Figure S4. The adverse prognostic association of *CEBPA* mutations persists across all six covariate adjustment levels, with an HR floor of 1.11 under maximum co-mutation adjustment. Supplementary Figure S5. *CEBPA* mutations retain adverse prognostic significance within the MDS-EB subgroup after IPSS-R adjustment. Supplementary Figure S6. *CEBPA* mutations in MDS arise across a broad VAF range, with truncating mutations enriched at subclonal allele frequencies. Supplementary Figure S7. Forest plot of stratum-specific hazard ratios for the association between *CEBPA* mutation status and overall survival across the 13 evaluable subgroups (unadjusted within stratum).
