# Supplementary material for: Clinical and Prognostic Significance of CEBPA Mutations in Myelodysplastic Syndromes
Source: Cancers (Basel). 2026 Jul 1;18(13):2135. doi: 10.3390/cancers18132135 (PMC13359441; doi:10.3390/cancers18132135)
Supplement: Supplementary file 1 [file cancers-18-02135-s001.zip › cancers-4391865-supplementary.pdf]

Table S1. Pre-specified sensitivity analyses: *CEBPA* Model B hazard ratio across nine analytic scenarios.

Analysis	n (total)	n *CEBPA*	OS events	*CEBPA* events	HR (95% CI)	p-value	Δ vs Reference
S0 - Reference: full cohort, Model B	2,216	59	1,073	36	1.39 (1.00–1.94)	0.053	-
S1 - Exclude OS < 1 month	2,151	59	1,054	36	1.43 (1.02–1.99)	0.037	stronger (+0.04)
S2 - Primary MDS only (exclude s/t-MDS)	1,952	56	909	33	1.42 (1.00–2.01)	0.049	stronger (+0.03)
S3 - Complete IPSS-R data only	2,216	59	1,073	36	1.39 (1.00–1.94)	0.053	~unchanged
S4 - IPSS-M–complete subsample (Model B)	2,195	56	1,060	34	1.38 (0.98–1.94)	0.068	~unchanged
S5 - Landmark analysis at 3 months	1,998	55	988	35	1.53 (1.09–2.14)	0.014	stronger (+0.14)
S6 - Competing risk: AML transformation	2,096	55	685	16	0.97 (0.59–1.59)	0.890	weaker (-0.42)
Co-mutation adjusted models							
S7 - Model B + *ASXL1* co-mutation adjustment	2,216	59	1,073	36	1.17 (0.83–1.64)	0.368	attenuated (-0.22)
S8 - Model B + *STAG2* co-mutation adjustment	2,216	59	1,073	36	1.22 (0.87–1.71)	0.259	attenuated (-0.17)
S9 - Model B + *ASXL1* + *STAG2* (most conservative)	2,216	59	1,073	36	1.11 (0.79–1.57)	0.540	attenuated (-0.28)

S0 (reference): full cohort, Model B (*CEBPA* + age + sex + IPSS-R score). S1: exclude OS <1 month. S2: primary MDS only. S3: complete IPSS-R data only (identical to S0 by construction). S4: IPSS-M-complete subsample, Model B. S5: landmark analysis at 3 months. S6: competing-risk analysis (AML transformation as event; death without transformation as competing event; cause-specific Cox). S7: Model B + *ASXL1*. S8: Model B + *STAG2*. S9: Model B + *ASXL1* + *STAG2* (most conservative adjustment).

CI, confidence interval; HR, hazard ratio; IPSS-M, Molecular International Prognostic Scoring System; IPSS-R, Revised International Prognostic Scoring System; MDS, myelodysplastic syndromes; OS, overall survival.

Table S2. *CEBPA* mutation subtype power analysis and descriptive characterisation.

Part A - Power Analysis

Comparison	Group	n	OS events	Threshold	Decision
Single-hit vs multi-hit	Multi-hit	10	8	≥10	- Underpowered (constrained per protocol)
Single-hit vs multi-hit	Single-hit	56	34	≥10	- Underpowered (constrained per protocol)
Truncating vs non-truncating	Truncating	36	29	≥10	✓ POWERED
Truncating vs non-truncating	Non-truncating	30	13	≥10	✓ POWERED
bZIP domain mutated vs not	bZIP-positive	31	23	≥10	✓ POWERED
bZIP domain mutated vs not	bZIP-negative	35	19	≥10	✓ POWERED
1 mutation vs 2+	1 mutation	56	34	≥10	- Underpowered
1 mutation vs 2+	2+ mutations	10	8	≥10	- Underpowered

Part B - Subtype Descriptives

Section	Group	n	% of *CEBPA*-mutated	OS events	Median OS (months)	HR (95% CI)	p-value	Note
Multi-hit vs single-hit (DESCRIPTIVE ONLY)								
	Multi-hit	10	15.2	8.0	18.4	NOT REPORTED	NOT REPORTED	Descriptive only: multi-hit n=10 events=8 - underpowered (<10 events); formal comparison not performed
	Single-hit	56	84.8	34.0	17.2	NOT REPORTED	NOT REPORTED	Reference group
Underpowered comparison: 1 mutation vs 2+ mutations								
	1 mutation	56		34.0	17.2	NOT REPORTED	NOT REPORTED	2+ mutations arm: n=10, events=8 - underpowere

								d (<10 events). Note: all n=2+ patients are multi-hit (phasing not confirmed).
	2+ mutations	10		8.0	18.4	NOT REPORTED	NOT REPORTED	
Mutation type (patient-level)								
	Has truncating mutation(s)	36	54.5					patient-level (not mutually exclusive)
	Has bZIP domain mutation(s)	31	47.0					patient-level (not mutually exclusive)
	Has N-terminal (TAD1) mutation(s)	40	60.6					patient-level (not mutually exclusive)
Mutation class (per mutation, not per patient)								
	Frameshift (Del + Ins)	41	53.9					% of 76 total mutation instances
	Nonsense	2	2.6					% of 76 total mutation instances
	Missense	29	38.2					% of 76 total mutation instances
	In-frame (Del + Ins)	4	5.3					% of 76 total mutation instances
Mutation type (raw, per mutation instance)								
	Missense_Mutation	29	38.2					% of 76 total mutation instances
	Frame_Shift_Ins	22	28.9					% of 76 total mutation instances
	Frame_Shift_Del	19	25.0					% of 76 total mutation instances
	In_Frame_Del	3	3.9					% of 76 total mutation instances
	Nonsense_Mutation	2	2.6					% of 76 total mutation instances

	In_Frame_Ins	1	1.3	% of 76 total mutation instances
Domain distribution (per mutation instance)				
	bZIP	32	48.5	% of *CEBPA*-mutated patients with >= 1 mutation in this domain
	TAD1	22	33.3	% of *CEBPA*-mutated patients with >= 1 mutation in this domain
	TAD2	20	30.3	% of *CEBPA*-mutated patients with >= 1 mutation in this domain
	DBD	2	3.0	% of *CEBPA*-mutated patients with >= 1 mutation in this domain

Part A: Pre-specified power screening based on minimum 10 OS events per arm. Multi-hit comparisons were designated descriptive per protocol due to insufficient events (multi-hit n=10; events=8). Part B: Descriptive summary of mutation subtypes; formal survival comparisons restricted to powered strata (truncating vs non-truncating; bZIP vs non-bZIP).

bZIP, basic leucine zipper domain; OS, overall survival; TAD, transactivation domain.

Table S3. Cross-cohort *CEBPA* mutation characterisation: IWG 2022 versus MSK-IMPACT 2020.

Section	Metric	IWG 2022	MSK 2020	p-value	Note
1. Mutation Frequency					
	Cohort size (n patients)	2442	977	-	MSK-filtered: MDS-specific samples only
	CEBPA-mutated (n)	66	14	-	
	CEBPA mutation rate (%)	2.7%	1.4%	0.026	IWG vs MSK-filtered (MDS-specific)
	MSK-full *CEBPA* rate (all hematologic)	2.7% (IWG ref)	4.6%	-	MSK-full n=4231 includes Papaemmanuil MDS 2013 + MSKCC-IMPACT + BeatAML; *CEBPA*=193
2. Mutation Type Distribution					
	Total mutations analysed	76	232	-	Mutation-level comparison (not patient-level)
	Truncating	43 (56.6%)	102 (44.0%)	-	
	Missense	29 (38.2%)	65 (28.0%)	-	
	In-frame	4 (5.3%)	65 (28.0%)	-	
	Chi-square (Truncating/Missense/In-frame)	-	-	<0.001	3-category test across broad mutation groups
	--- Granular Variant Classification ---	IWG n (%)	MSK n (%)	-	
	Frame_Shift_Del	19 (25.0%)	25 (10.8%)	-	
	Frame_Shift_Ins	22 (28.9%)	60 (25.9%)	-	
	In_Frame_Del	3 (3.9%)	1 (0.4%)	-	
	In_Frame_Ins	1 (1.3%)	64 (27.6%)	-	
	Missense_Mutation	29 (38.2%)	65 (28.0%)	-	
	Nonsense_Mutation	2 (2.6%)	14 (6.0%)	-	
	Nonstop_Mutation	0 (0.0%)	1 (0.4%)	-	
	Splice_Site	0 (0.0%)	2 (0.9%)	-	
3. Domain Distribution					
	Total mutations analysed	76	232	-	
	bZIP	32 (42.1%)	134 (57.8%)	-	
	TAD1	22 (28.9%)	70 (30.2%)	-	

Section	Metric	IWG 2022	MSK 2020	p-value	Note
	TAD2	20 (26.3%)	22 (9.5%)	-	
	DBD	2 (2.6%)	6 (2.6%)	-	
	Other	0 (0.0%)	0 (0.0%)	-	
	Chi-square (bZIP/TAD1/TAD2/DBD)	-	-	0.002	4 main domains; Other excluded from test
4. Single-hit vs Multi-hit CEBPA					
	Total *CEBPA*-mutated patients	66	193	-	MSK = MSK-full detail (193 patients)
	Single-hit (1 mutation)	56 (84.8%)	156 (80.8%)	-	
	Multi-hit (2+ mutations)	10 (15.2%)	37 (19.2%)	-	
	Statistical test	-	-	0.465	Single-hit vs multi-hit proportion comparison
5. Top Co-mutated Genes in CEBPA-mutated Patients					
	Denominator (*CEBPA*-mutated patients)	66 (IWG final cohort)	193 (MSK-full detail)	-	Frequencies = % of *CEBPA*-mutated patients with mutation
	Note on panel differences	IWG: error-corrected sequencing panel ~200-400 genes	MSK: MSK-IMPACT (341/410/468 genes) + Papaemmanuil WES	-	Panel differences may explain gene frequency discordance
	--- Gene-level comparison ---	IWG: n (%) [FDR sig]	MSK: n (%)	-	
	ASXL1	42 (63.6%) [FDR*]	15 (7.8%)	-	
	STAG2	30 (45.5%) [FDR*]	13 (6.7%)	-	
	SRSF2	25 (37.9%) [FDR*]	18 (9.3%)	-	
	TET2	25 (37.9%)	42 (21.8%)	-	
	RUNX1	18 (27.3%) [FDR*]	16 (8.3%)	-	
	BCOR	14 (21.2%) [FDR*]	0 (0.0%)	-	
	SF3B1	10 (15.2%)	0 (0.0%)	-	
	DNMT3A	10 (15.2%)	29 (15.0%)	-	
	IDH2	9 (13.6%) [FDR*]	14 (7.3%)	-	
	U2AF1	7 (10.6%)	0 (0.0%)	-	

Section	Metric	IWG 2022	MSK 2020	p-value	Note
	EZH2	7 (10.6%)	13 (6.7%)	-	
	JAK2	5 (7.6%)	0 (0.0%)	-	
	MPL	5 (7.6%)	0 (0.0%)	-	
	ZRSR2	5 (7.6%)	0 (0.0%)	-	
	WT1	4 (6.1%)	25 (13.0%)	-	
	FLT3	0 (0.0%)	11 (5.7%)	-	
	GATA2	0 (0.0%)	36 (18.7%)	-	
	RAD21	0 (0.0%)	9 (4.7%)	-	
	NRAS	0 (0.0%)	23 (11.9%)	-	
	NPM1	0 (0.0%)	19 (9.8%)	-	
	TP53	0 (0.0%)	17 (8.8%)	-	
	Genes FDR-significant in IWG (all)	6	Not computed for MSK (no WT comparison available)	-	FDR-significant = enriched in *CEBPA* vs WT, FDR<0.05

MSK cohort filtered to MDS-specific Oncotree codes (n=977) for frequency comparison. Survival endpoints not available in MSK cohort. Gene-level co-mutation frequencies reflect *CEBPA*-mutated patients only; wild-type comparison not available for MSK due to absence of matched MDS-specific controls. FDR significance [FDR*] refers to IWG cohort enrichment analysis (Supplementary Table 4).

CEBPA, CCAAT/enhancer-binding protein alpha; IWG, International Working Group; MDS, myelodysplastic syndromes; MSK, Memorial Sloan Kettering.

Table S4. Co-mutation enrichment analysis: gene-level co-occurrence in *CEBPA*-mutated versus wild-type MDS patients (IWG 2022 cohort, n=2,442).

Gene	*CEBPA*-mut n (%)	Wild-type n (%)	Odds ratio	p-value	FDR q-value	FDR significant	Association direction
ASXL1	42 (63.6%)	600 (25.2%)	5.18	<0.001	<0.001	Yes	Co Occurrence
STAG2	30 (45.5%)	204 (8.6%)	8.87	<0.001	<0.001	Yes	Co Occurrence
SRSF2	25 (37.9%)	327 (13.8%)	3.82	<0.001	<0.001	Yes	Co Occurrence
TET2	25 (37.9%)	681 (28.7%)	1.52	0.129	0.391		Co Occurrence
RUNX1	18 (27.3%)	307 (12.9%)	2.53	0.003	0.043	Yes	Co Occurrence
BCOR	14 (21.2%)	152 (6.4%)	3.94	<0.001	0.002	Yes	Co Occurrence
DNMT3A	10 (15.2%)	410 (17.3%)	0.86	0.743	0.929		Mutual Exclusivity
SF3B1	10 (15.2%)	597 (25.1%)	0.53	0.082	0.390		Mutual Exclusivity
IDH2	9 (13.6%)	107 (4.5%)	3.35	0.003	0.048	Yes	Co Occurrence
EZH2	7 (10.6%)	157 (6.6%)	1.68	0.207	0.533		Co Occurrence
U2AF1	7 (10.6%)	226 (9.5%)	1.13	0.674	0.909		Co Occurrence
ZRSR2	5 (7.6%)	137 (5.8%)	1.34	0.432	0.794		Co Occurrence
JAK2	5 (7.6%)	78 (3.3%)	2.42	0.071	0.390		Co Occurrence
MPL	5 (7.6%)	69 (2.9%)	2.74	0.047	0.336		Co Occurrence
WT1	4 (6.1%)	45 (1.9%)	3.34	0.041	0.336		Co Occurrence
NRAS	4 (6.1%)	78 (3.3%)	1.90	0.281	0.645		Co Occurrence
NOTCH1	4 (6.1%)	95 (4.0%)	1.55	0.341	0.715		Co Occurrence
SH2B3	4 (6.1%)	66 (2.8%)	2.26	0.118	0.391		Co Occurrence
EP300	4 (6.1%)	86 (3.6%)	1.72	0.305	0.682		Co Occurrence
SETBP1	4 (6.1%)	127 (5.3%)	1.14	0.779	0.946		Co Occurrence
ASXL2	3 (4.5%)	47 (2.0%)	2.36	0.151	0.443		Co Occurrence
CSF3R	3 (4.5%)	42 (1.8%)	2.65	0.120	0.391		Co Occurrence
ZNF318	3 (4.5%)	83 (3.5%)	1.32	0.504	0.796		Co Occurrence
NF1	3 (4.5%)	112 (4.7%)	0.96	1.000	1.000		Mutual Exclusivity
RAD21	3 (4.5%)	30 (1.3%)	3.72	0.058	0.378		Co Occurrence
YLPM1	3 (4.5%)	82 (3.5%)	1.33	0.499	0.796		Co Occurrence
ZBTB33	3 (4.5%)	37 (1.6%)	3.01	0.092	0.390		Co Occurrence
SPRED2	3 (4.5%)	22 (0.9%)	5.09	0.028	0.268		Co Occurrence
GNAS-AS1	3 (4.5%)	40 (1.7%)	2.78	0.108	0.391		Co Occurrence
DHX33	3 (4.5%)	27 (1.1%)	4.14	0.046	0.336		Co Occurrence

Gene	*CEBPA*-mut n (%)	Wild-type n (%)	Odds ratio	p-value	FDR q-value	FDR significant	Association direction
CBL	3 (4.5%)	109 (4.6%)	0.99	1.000	1.000		Mutual Exclusivity
CUX1	3 (4.5%)	130 (5.5%)	0.82	1.000	1.000		Mutual Exclusivity
BCL10	2 (3.0%)	3 (0.1%)	24.72	0.007	0.083		Co Occurrence
ARID2	2 (3.0%)	61 (2.6%)	1.19	0.687	0.909		Co Occurrence
CREBBP	2 (3.0%)	74 (3.1%)	0.97	1.000	1.000		Mutual Exclusivity
DNMT3B	2 (3.0%)	50 (2.1%)	1.45	0.650	0.909		Co Occurrence
C7orf55-LUC7L2	2 (3.0%)	20 (0.8%)	3.68	0.118	0.391		Co Occurrence
RRAS	2 (3.0%)	17 (0.7%)	4.34	0.092	0.390		Co Occurrence
STAT5A	2 (3.0%)	28 (1.2%)	2.62	0.194	0.514		Co Occurrence
U2AF2	2 (3.0%)	19 (0.8%)	3.88	0.109	0.391		Co Occurrence
TP53	2 (3.0%)	309 (13.0%)	0.21	0.013	0.142		Mutual Exclusivity
SMC1A	2 (3.0%)	28 (1.2%)	2.62	0.194	0.514		Co Occurrence
SETD2	2 (3.0%)	58 (2.4%)	1.25	0.676	0.909		Co Occurrence
SMG1	2 (3.0%)	73 (3.1%)	0.99	1.000	1.000		Mutual Exclusivity
ROBO2	2 (3.0%)	63 (2.6%)	1.15	0.695	0.909		Co Occurrence
NXF1	2 (3.0%)	18 (0.8%)	4.09	0.100	0.391		Co Occurrence
NF2	2 (3.0%)	16 (0.7%)	4.61	0.083	0.390		Co Occurrence
MGA	2 (3.0%)	99 (4.2%)	0.72	1.000	1.000		Mutual Exclusivity
KRAS	2 (3.0%)	52 (2.2%)	1.40	0.656	0.909		Co Occurrence
KMT2C	2 (3.0%)	123 (5.2%)	0.57	0.773	0.946		Mutual Exclusivity
KDM6A	2 (3.0%)	43 (1.8%)	1.70	0.345	0.715		Co Occurrence
IDH1	2 (3.0%)	81 (3.4%)	0.89	1.000	1.000		Mutual Exclusivity
GATA1	2 (3.0%)	16 (0.7%)	4.61	0.083	0.390		Co Occurrence
NPM1	2 (3.0%)	28 (1.2%)	2.62	0.194	0.514		Co Occurrence
PRPF40B	2 (3.0%)	42 (1.8%)	1.74	0.335	0.715		Co Occurrence
DDX4	1 (1.5%)	22 (0.9%)	1.65	0.469	0.796		Co Occurrence
DDX41	1 (1.5%)	90 (3.8%)	0.39	0.516	0.796		Mutual Exclusivity
BCORL1	1 (1.5%)	64 (2.7%)	0.56	1.000	1.000		Mutual Exclusivity
CDKN2B	1 (1.5%)	4 (0.2%)	9.12	0.128	0.391		Co Occurrence
ARID1A	1 (1.5%)	74 (3.1%)	0.48	0.721	0.918		Mutual Exclusivity
BAP1	1 (1.5%)	10 (0.4%)	3.64	0.261	0.615		Co Occurrence

Gene	*CEBPA*-mut n (%)	Wild-type n (%)	Odds ratio	p-value	FDR q-value	FDR significant	Association direction
EED	1 (1.5%)	17 (0.7%)	2.13	0.390	0.737		Co Occurrence
CSNK1A1	1 (1.5%)	39 (1.6%)	0.92	1.000	1.000		Mutual Exclusivity
FAM175A	1 (1.5%)	17 (0.7%)	2.13	0.390	0.737		Co Occurrence
ETV6	1 (1.5%)	55 (2.3%)	0.65	1.000	1.000		Mutual Exclusivity
IRF1	1 (1.5%)	22 (0.9%)	1.65	0.469	0.796		Co Occurrence
FLT3	1 (1.5%)	32 (1.4%)	1.13	0.598	0.876		Co Occurrence
ETNK1	1 (1.5%)	63 (2.6%)	0.56	1.000	1.000		Mutual Exclusivity
KIT	1 (1.5%)	38 (1.6%)	0.95	1.000	1.000		Mutual Exclusivity
PHIP	1 (1.5%)	62 (2.6%)	0.57	1.000	1.000		Mutual Exclusivity
PHF6	1 (1.5%)	79 (3.3%)	0.45	0.723	0.918		Mutual Exclusivity
PAX5	1 (1.5%)	8 (0.3%)	4.55	0.219	0.547		Co Occurrence
PAPD5	1 (1.5%)	15 (0.6%)	2.42	0.356	0.720		Co Occurrence
KMT2D	1 (1.5%)	168 (7.1%)	0.20	0.085	0.390		Mutual Exclusivity
HIPK2	1 (1.5%)	23 (1.0%)	1.57	0.484	0.796		Co Occurrence
JARID2	1 (1.5%)	31 (1.3%)	1.16	0.586	0.874		Co Occurrence
RB1	1 (1.5%)	34 (1.4%)	1.06	0.619	0.892		Co Occurrence
SMC3	1 (1.5%)	26 (1.1%)	1.39	0.525	0.796		Co Occurrence
SF1	1 (1.5%)	20 (0.8%)	1.81	0.439	0.794		Co Occurrence
PRPF8	1 (1.5%)	53 (2.2%)	0.67	1.000	1.000		Mutual Exclusivity
RAD50	1 (1.5%)	38 (1.6%)	0.95	1.000	1.000		Mutual Exclusivity
TERT	1 (1.5%)	9 (0.4%)	4.05	0.240	0.583		Co Occurrence
SUZ12	1 (1.5%)	25 (1.1%)	1.45	0.511	0.796		Co Occurrence
SRCAP	1 (1.5%)	118 (5.0%)	0.29	0.375	0.737		Mutual Exclusivity
STAG1	1 (1.5%)	25 (1.1%)	1.45	0.511	0.796		Co Occurrence

Shaded rows indicate Benjamini-Hochberg FDR-significant enrichment (q<0.05). Genes with expected cell counts <5 were tested by Fisher's exact test; others by chi-squared test. Multiple comparisons corrected by Benjamini-Hochberg FDR at q<0.05 threshold. Only genes present in ≥2 *CEBPA*-mutated patients shown.

CI, confidence interval; FDR, false discovery rate; OR, odds ratio.

Table S5. Univariate Cox proportional hazards regression for all tested variables - overall survival and leukaemia-free survival.

Endpoint	Variable	n	Events	HR	95% CI	p-value	Concordance
Overall Survival							
	CEBPA status (mutated vs WT)	2,442	1,207	2.05	1.50–2.79	<0.001	0.510
	Age (per year)	2,440	1,206	1.02	1.02–1.03	<0.001	0.549
	Sex (Male vs Female)	2,442	1,207	1.30	1.15–1.46	<0.001	0.533
	WHO 2016 (vs MDS-MLD) - MDS-EB1 vs MDS-MLD	2,381	1,176	1.79	1.50–2.12	<0.001	0.646
	WHO 2016 (vs MDS-MLD) - MDS-EB2 vs MDS-MLD	2,381	1,176	2.41	2.03–2.85	<0.001	0.646
	WHO 2016 (vs MDS-MLD) - MDS-del5q vs MDS-MLD	2,381	1,176	0.73	0.54–0.97	0.030	0.646
	MDS type (s/t-MDS vs primary)	2,355	1,158	1.64	1.37–1.96	<0.001	0.527
	BM blast % (per unit)	2,373	1,166	1.08	1.07–1.10	<0.001	0.633
	PB blast % (per unit)	1,987	989	1.13	1.10–1.16	<0.001	0.554
	Hemoglobin (per g/dL)	2,376	1,174	0.84	0.82–0.87	<0.001	0.612
	Platelet count (per x10^9/L)	2,365	1,170	1.00	1.00–1.00	<0.001	0.627
	WBC (per x10^9/L)	2,248	1,095	0.97	0.95–1.00	0.024	0.565
	ANC (per x10^9/L)	2,323	1,143	0.95	0.92–0.98	0.002	0.570
	IPSS-R category (ordinal)	2,302	1,129	1.71	1.63–1.80	<0.001	0.696
	IPSS-R score (per unit)	2,217	1,074	1.41	1.37–1.45	<0.001	0.706
	IPSS-M category (ordinal)	2,231	1,084	1.60	1.55–1.66	<0.001	0.736
	IPSS-M score (per unit)	2,231	1,084	1.78	1.71–1.86	<0.001	0.749
	Cytogenetic risk (ordinal)	2,334	1,136	1.68	1.59–1.78	<0.001	0.615
	Complex karyotype (vs non-complex)	2,442	1,207	4.24	3.62–4.96	<0.001	0.578
Leukemia-Free Survival							
	CEBPA status (mutated vs WT)	2,301	1,195	1.79	1.30–2.48	<0.001	0.507
	Age (per year)	2,300	1,194	1.02	1.01–1.02	<0.001	0.533

Endpoint	Variable	n	Events	HR	95% CI	p-value	Concordance
	Sex (Male vs Female)	2,301	1,195	1.32	1.17–1.48	<0.001	0.535
	WHO 2016 (vs MDS-MLD) - MDS-EB1 vs MDS-MLD	2,257	1,171	1.83	1.54–2.17	<0.001	0.658
	WHO 2016 (vs MDS-MLD) - MDS-EB2 vs MDS-MLD	2,257	1,171	2.66	2.25–3.15	<0.001	0.658
	WHO 2016 (vs MDS-MLD) - MDS-del5q vs MDS-MLD	2,257	1,171	0.77	0.57–1.02	0.068	0.658
	MDS type (s/t-MDS vs primary)	2,231	1,150	1.60	1.34–1.91	<0.001	0.526
	BM blast % (per unit)	2,246	1,160	1.10	1.09–1.11	<0.001	0.649
	PB blast % (per unit)	1,882	987	1.12	1.09–1.16	<0.001	0.552
	Hemoglobin (per g/dL)	2,247	1,168	0.86	0.84–0.89	<0.001	0.597
	Platelet count (per x10^9/L)	2,236	1,164	1.00	1.00–1.00	<0.001	0.621
	WBC (per x10^9/L)	2,129	1,095	0.97	0.95–0.99	0.008	0.570
	ANC (per x10^9/L)	2,197	1,138	0.92	0.89–0.96	<0.001	0.578
	IPSS-R category (ordinal)	2,175	1,122	1.72	1.64–1.80	<0.001	0.697
	IPSS-R score (per unit)	2,097	1,072	1.40	1.36–1.44	<0.001	0.708
	IPSS-M category (ordinal)	2,113	1,083	1.62	1.56–1.69	<0.001	0.738
	IPSS-M score (per unit)	2,113	1,083	1.80	1.73–1.87	<0.001	0.751
	Cytogenetic risk (ordinal)	2,200	1,128	1.64	1.55–1.73	<0.001	0.614
	Complex karyotype (vs non-complex)	2,301	1,195	3.90	3.32–4.57	<0.001	0.575

All tests are univariate (unadjusted). *CEBPA* mutation status is listed first for reference. Concordance index (C-statistic) reflects the predictive discrimination of each variable individually.

ANC, absolute neutrophil count; BM, bone marrow; CI, confidence interval; HR, hazard ratio; IPSS-M, Molecular International Prognostic Scoring System; IPSS-R, Revised International Prognostic Scoring System; LFS, leukaemia-free survival; OS, overall survival; PB, peripheral blood; WBC, white blood cell count; WHO, World Health Organization.

Table S6. Cox proportional hazards regression restricted to the MDS-EB subcohort (MDS-EB1 + MDS-EB2; n=870; OS events=562).

Model	Covariate	n	Events	HR	95% CI	p-value	Schoenfeld p
Overall Survival							
**Univariate**							
	CEBPA mutation (mutated vs WT)	870	562	1.53	1.07–2.19	0.020	-
Leukemia-Free Survival							
**Univariate**							
	CEBPA mutation (mutated vs WT)	836	569	1.31	0.91–1.90	0.150	-
Overall Survival							
**Model B (*CEBPA* + Age + Sex + IPSS-R)**							
	CEBPA mutation (mutated vs WT)	811	519	1.48	1.02–2.14	0.038	0.270
	Age (per year)	811	519	1.03	1.02–1.04	<0.001	0.053
	Sex (male vs female)	811	519	1.06	0.88–1.27	0.518	0.190
	IPSS-R score (per unit)	811	519	1.46	1.38–1.54	<0.001	0.005
Leukemia-Free Survival							
**Model B (*CEBPA* + Age + Sex + IPSS-R)**							
	CEBPA mutation (mutated vs WT)	779	527	1.26	0.86–1.85	0.232	0.293
	Age (per year)	779	527	1.02	1.01–1.03	<0.001	0.002
	Sex (male vs female)	779	527	1.11	0.93–1.33	0.251	0.128
	IPSS-R score (per unit)	779	527	1.38	1.31–1.46	<0.001	0.008

MDS-EB subcohort: WHO 2016 classification MDS-EB1 or MDS-EB2. Model B: *CEBPA* + age (continuous) + sex + IPSS-R score (continuous); restricted to patients with complete covariate data (OS n=811, events=519; LFS n=779, events=527). Schoenfeld p: proportional hazards test (rank-transform); reported for OS Model B only. *CEBPA*: Schoenfeld p=0.270 (assumption satisfied).

CI, confidence interval; HR, hazard ratio; IPSS-R, Revised International Prognostic Scoring System; MDS-EB, MDS with excess blasts; OS, overall survival; LFS, leukaemia-free survival.

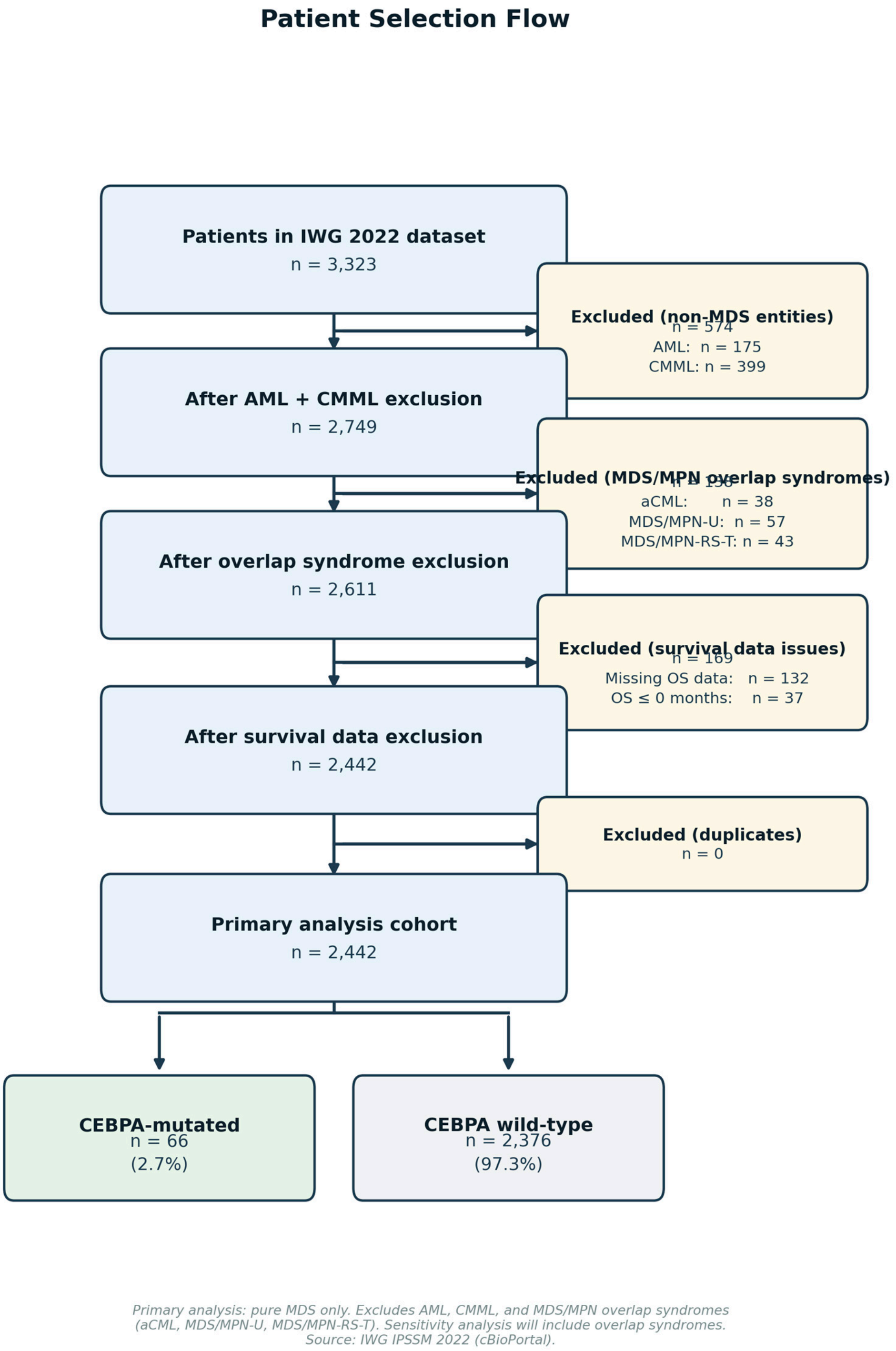

Figure S1. *CEBPA* mutations were identified in 66 of 2,442 evaluable MDS patients (2.7%) after sequential pre-specified diagnostic exclusions.

Starting from 3,323 patients in the IWG 2022 MDS registry, exclusions were applied sequentially: AML (bone marrow blasts ≥20%; n=175), CMML (n=399), MDS/MPN overlap syndromes including atypical CML, MDS/MPN unclassifiable, and MDS/MPN-RS-T (n=138 combined), missing OS status or duration (n=132), and OS ≤0 months (n=37). The final evaluable cohort comprised 2,442 patients: *CEBPA*-mutated n=66 (2.7%), *CEBPA* wild-type n=2,376 (97.3%).

AML, acute myeloid leukaemia; CMML, chronic myelomonocytic leukaemia; CML, chronic myeloid leukaemia; IWG, International Working Group; MDS, myelodysplastic syndromes; MPN, myeloproliferative neoplasm; MPN-RS-T, MPN with ring sideroblasts and thrombocytosis; OS, overall survival.

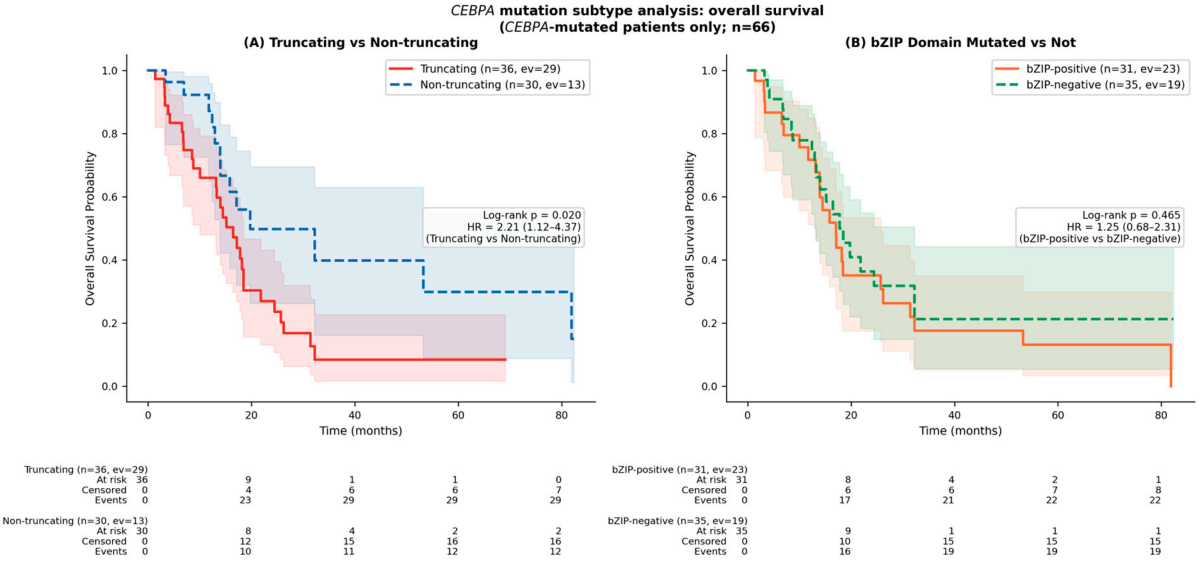

Figure S2. Truncating *CEBPA* mutations drive the adverse survival signal in MDS; bZIP domain mutations confer no detectable survival effect.

Kaplan-Meier OS curves within *CEBPA*-mutated patients; tick marks indicate censored observations. **(A)** Truncating (n=36; 29 events) versus non-truncating (n=30; 13 events): HR 2.21 (95% CI 1.12–4.37; p=0.023). **(B)** bZIP domain-involving (n=31; 23 events) versus non-bZIP (n=35; 19 events): HR 1.25 (95% CI 0.68–2.31; p=0.470).

bZIP, basic leucine zipper; CI, confidence interval; HR, hazard ratio; MDS, myelodysplastic syndromes; OS, overall survival.

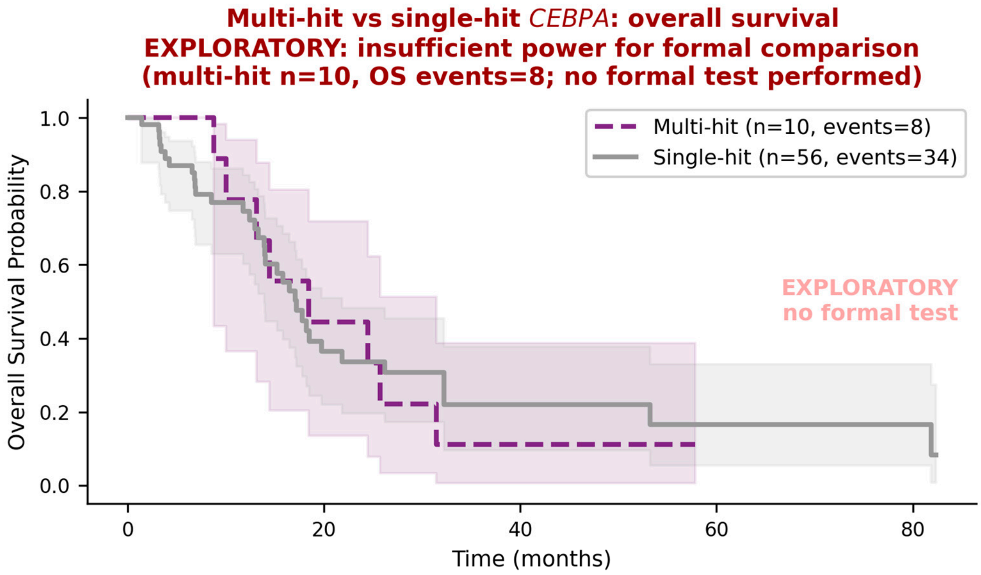

**Multi-hit (n=10, events=8)**							
At risk	0	10	4	1	0	0	0
Censored	0	0	1	1	2	2	2
Events	0	0	5	8	8	8	8
**Single-hit (n=56, events=34)**							
At risk	0	56	13	4	3	2	0
Censored	0	0	15	20	20	21	22
Events	0	0	28	32	33	33	34

Figure S3. Multi-hit *CEBPA* mutations do not confer survival benefit in MDS - descriptive comparison, no formal test.

Kaplan-Meier OS curves for multi-hit (n=10; median OS 18.4 months) versus single-hit *CEBPA* mutation (n=56; median OS 17.2 months). Vertical tick marks indicate censored observations. **Formal comparison not performed - insufficient power (n=10 multi-hit patients, 8 events).** This contrasts with AML, where biallelic *CEBPA* mutations define European LeukemiaNet Favourable risk with five-year OS approaching 60%.

AML, acute myeloid leukaemia; MDS, myelodysplastic syndromes; OS, overall survival.

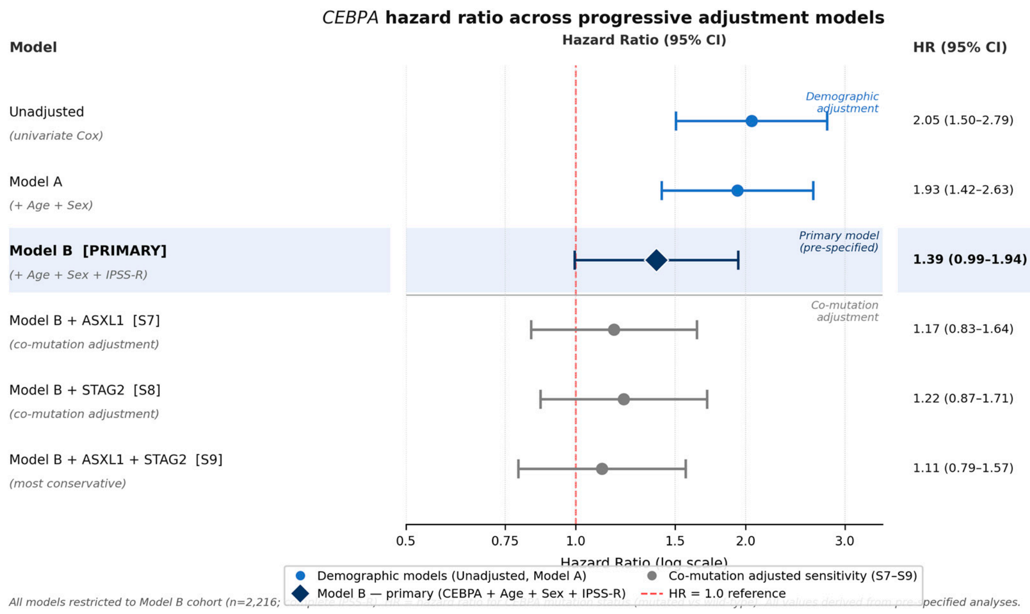

Figure S4. The adverse prognostic association of *CEBPA* mutations persists across all six covariate adjustment levels, with an HR floor of 1.11 under maximum co-mutation adjustment.

Horizontal forest plot of the *CEBPA* Model B HR across six covariate adjustment levels: Unadjusted (HR 2.05), Model A (HR 1.93), Model B-Primary (HR 1.39), S7-Model B + *ASXL1* (HR 1.17), S8-Model B + *STAG2* (HR 1.22), and S9-Model B + *ASXL1* + *STAG2* (HR 1.11). The HR floor is 1.11 under maximum co-mutation adjustment.

CI, confidence interval; HR, hazard ratio.

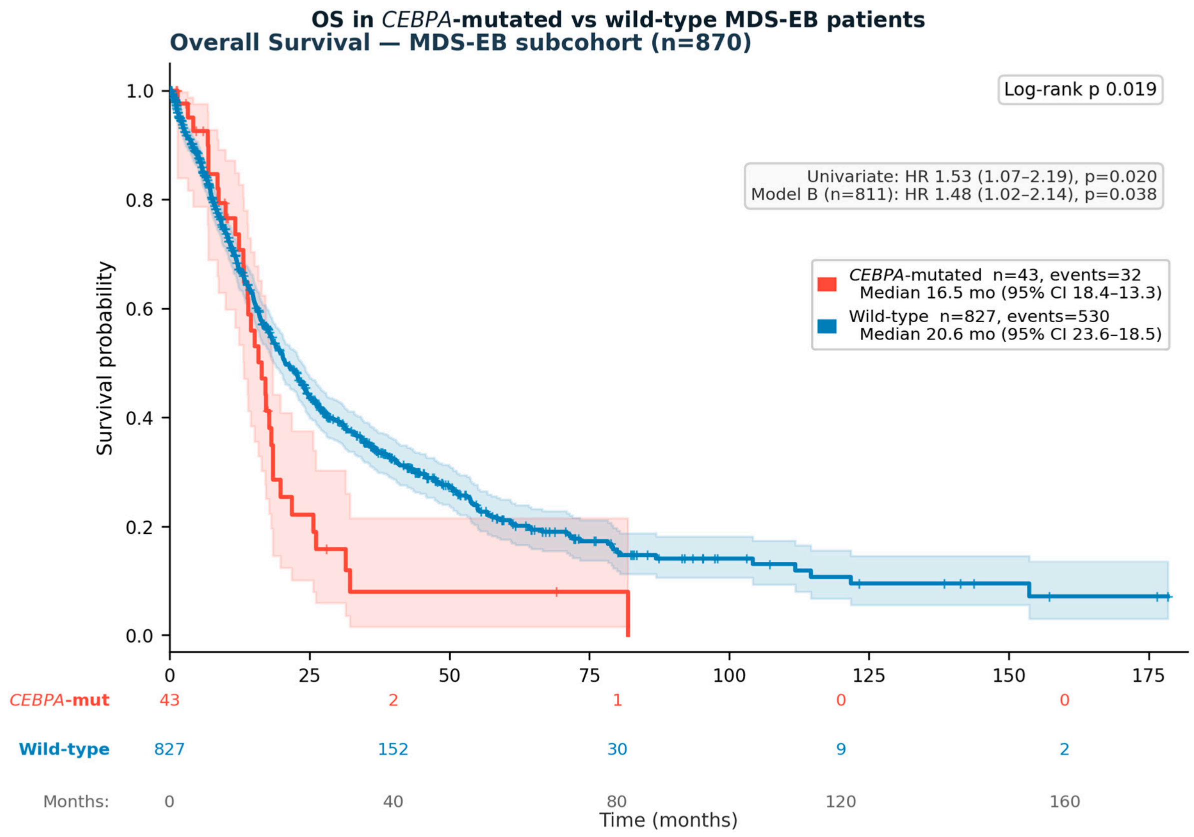

Figure S5. *CEBPA* mutations retain adverse prognostic significance within the MDS-EB subgroup after IPSS-R adjustment.

Kaplan-Meier OS curves for *CEBPA*-mutated (n=43; red) and wild-type patients (n=827; blue) restricted to MDS-EB1 and MDS-EB2 (n=870 total); vertical tick marks indicate censored observations. Univariate HR 1.53 (95% CI 1.07–2.19; p=0.02). Model B (adjusted for age, sex, IPSS-R score; n=811): HR 1.48 (95% CI 1.02–2.14; p=0.04). Number-at-risk table shown beneath panel.

CI, confidence interval; HR, hazard ratio; MDS-EB, MDS with excess blasts; OS, overall survival.

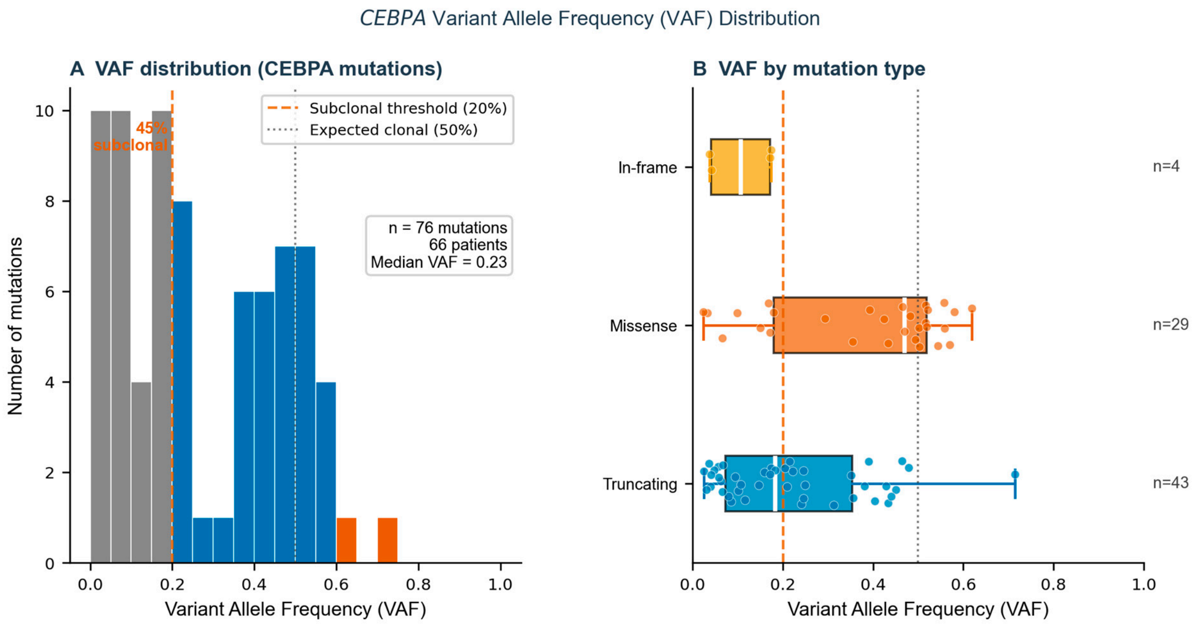

Figure S6. *CEBPA* mutations in MDS arise across a broad VAF range, with truncating mutations enriched at subclonal allele frequencies.

Variant allele frequencies (VAFs) of 76 *CEBPA* mutations in 66 IWG final-cohort patients. **(A)** VAF histogram; reference lines at the 20% subclonal threshold (dashed) and 50% expected clonal VAF (dotted); 44.7% (34/76) mutations fall below the subclonal threshold. **(B)** VAF stratified by mutation type: truncating mutations show markedly lower median VAF than missense mutations (0.18 versus 0.47).

IWG, International Working Group; MDS, myelodysplastic syndromes; VAF, variant allele frequency.

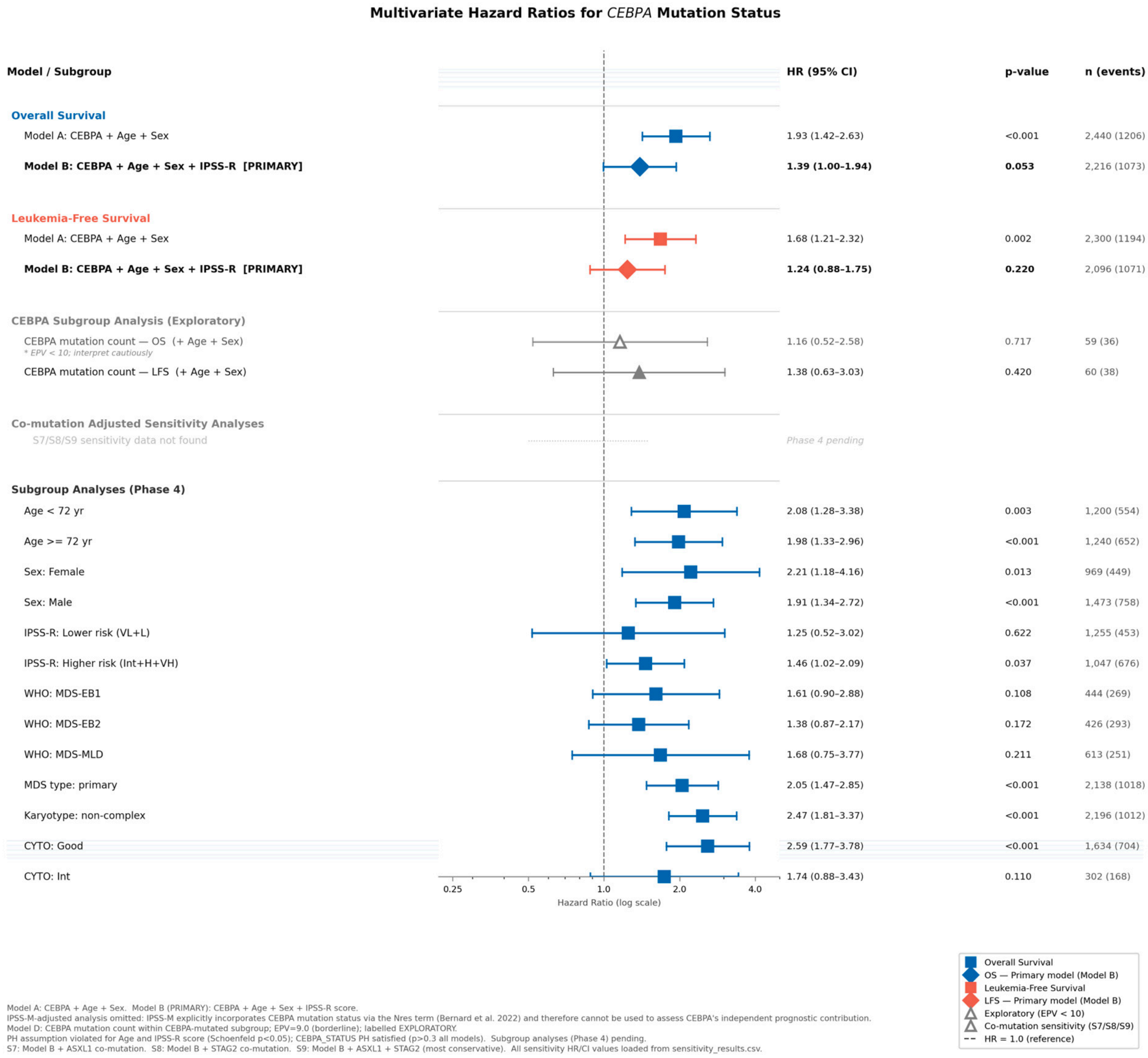

Figure S7. Forest plot of stratum-specific hazard ratios for the association between CEBPA mutation status and overall survival across the 13 evaluable subgroups (unadjusted within stratum).
